# Weyl nodal ring states and Landau quantization with very large magnetoresistance in square-net magnet EuGa_4_

**DOI:** 10.1038/s41467-023-40767-z

**Published:** 2023-09-19

**Authors:** Shiming Lei, Kevin Allen, Jianwei Huang, Jaime M. Moya, Tsz Chun Wu, Brian Casas, Yichen Zhang, Ji Seop Oh, Makoto Hashimoto, Donghui Lu, Jonathan Denlinger, Chris Jozwiak, Aaron Bostwick, Eli Rotenberg, Luis Balicas, Robert Birgeneau, Matthew S. Foster, Ming Yi, Yan Sun, Emilia Morosan

**Affiliations:** 1 Department of Physics and Astronomy, Rice University, Houston, TX 77005 USA; 2 Rice Center for Quantum Materials, Rice University, Houston, TX 77005 USA; 3 Applied Physics Graduate Program, Rice University, Houston, TX 77005 USA; 4 National High Magnetic Field Laboratory, Tallahase, FL 32310 USA; 5 Department of Physics, University of California, Berkeley, CA 94720 USA; 6 Stanford Synchrotron Radiation Lightsource, SLAC National Accelerator Laboratory, Menlo Park, CA 94025 USA; 7 Advanced Light Source, Lawrence Berkeley National Laboratory, Berkeley, CA 94720 USA; 8 Department of Physics, Florida State University, Tallahassee, FL 32306 USA; 9 Materials Science Division, Lawrence Berkeley National Laboratory, Berkeley, CA 94720 USA; 10 Shenyang National Laboratory for Materials Science, Institute of Metal Research, Chinese Academy of Sciences, Shenyang, 110016 China

**Keywords:** Topological matter, Materials science

## Abstract

Magnetic topological semimetals allow for an effective control of the topological electronic states by tuning the spin configuration. Among them, Weyl nodal line semimetals are thought to have the greatest tunability, yet they are the least studied experimentally due to the scarcity of material candidates. Here, using a combination of angle-resolved photoemission spectroscopy and quantum oscillation measurements, together with density functional theory calculations, we identify the square-net compound EuGa_4_ as a magnetic Weyl nodal ring semimetal, in which the line nodes form closed rings near the Fermi level. The Weyl nodal ring states show distinct Landau quantization with clear spin splitting upon application of a magnetic field. At 2 K in a field of 14 T, the transverse magnetoresistance of EuGa_4_ exceeds 200,000%, which is more than two orders of magnitude larger than that of other known magnetic topological semimetals. Our theoretical model suggests that the non-saturating magnetoresistance up to 40 T arises as a consequence of the nodal ring state.

## Abstract (web summary)

The tunability of electronic properties is a central goal of research into topological semimetals. Here, the authors report Weyl nodal ring states in the magnetic semimetal EuGa_4_ and link the nodal ring state to the observed large non-saturating magnetoresistance.

## Introduction

Magnetic topological semimetals (TSMs) that are characterized by linear-band crossings in momentum space have been established as hosts to many emergent properties, such as Fermi arc surface states^1^, the chiral anomaly^2,3^, large anomalous Hall effect (AHE)^4–7^ and drumhead surface states^8,9^. Compared to their nonmagnetic counterparts, magnetic TSMs provide a unique opportunity to tune their electronic structure and, consequently, the band topology by manipulating the spin configuration, thus providing an important materials platform for the design of topological electronic and spintronic devices^10–12^.

For magnetic TSMs, the band crossings can result in isolated points or lines, giving rise to Weyl points or Weyl nodal-line (NL) states, respectively. In principle, the formation of the former states requires only the lattice translation symmetry, while the latter demands additional symmetries such as a mirror reflection^13^. When the mirror reflection is destroyed, for example, by rotating the magnetic moments under an applied magnetic field, the Weyl NLs become gapped and Weyl point states emerge^14,15^.

Although there has been great progress in theoretical studies of Weyl NLs in magnetic TSMs^8,9,14,16–20^, their experimental realization is rather limited, especially in the presence of spin-orbit coupling (SOC)^11,12^. For example, in Fe_3_GeTe_2_ the NL states are gapped by SOC, although the gap is small at certain locations in the momentum space^21^. Thus far, only the Co-based Heusler alloys Co_2_MnZ (*Z* = Ga and Al)^9,22^ have been experimentally identified as magnetic Weyl NL semimetals, and only Co_2_MnGa has gained a good understanding of the electronic structure through spectroscopy measurements^9^. Nevertheless, magnetotransport properties in magnetic Weyl NL semimetals, particularly in the Landau quantized regime, where *μB* > 1 (*μ* is the carrier mobility and *B* is the applied magnetic field)^23,24^, are largely unexplored. It is imperative to experimentally identify new magnetic Weyl NL candidates, ideally with high carrier mobility, Weyl NL states close to the Fermi level *E*_*F*_, and small energy variation, to maximize their effects on the electronic properties^25–27^.

Here, we report the discovery of Weyl nodal ring (NR, or closed-loop NL) states near E_*F*_ in the magnetic square-net EuGa_4_ in the presence of mirror symmetry protection. Using angle-resolved photoemission spectroscopy (ARPES) and quantum oscillation (QO) measurements, we probe the electronic structures of EuGa_4_ in both the paramagnetic and spin-polarized (SP) states. The good agreement between experimental and density functional theory (DFT) calculation results provides strong evidence for the existence of Weyl NR states with low dispersion along the ring near *E*_*F*_. The quantum mobility is among the highest of all known magnetic TSMs. Associated with the Weyl NR states, we report very large, non-saturating transverse magnetoresistance (MR) up to the Landau quantized regime, exceeding 200,000 % at *T* = 2 K and *μ*_0_*H* = 14 T. This value is more than two orders of magnitude higher than that of other known magnetic TSMs, and comparable even with the higher values in nonmagnetic TSMs. Our magnetotransport theoretical model directly shows how the nodal ring states yield large non-saturating MR.

## Results

### Mechanism for Weyl NRs formation in a square lattice

**Fig. 1:**
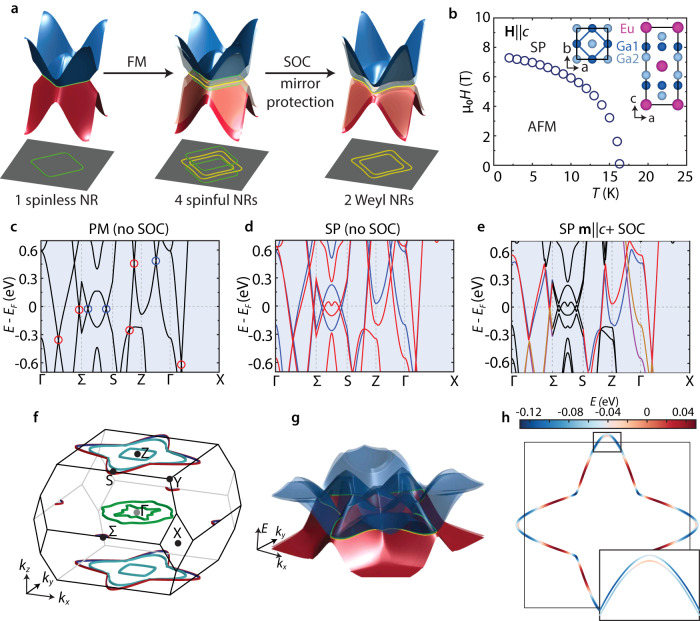
EuGa_4_ as a candidate to host Weyl NR states. **a** The proposed mechanism to create Weyl NR states in square-net magnetic materials: one spinless NR evolves into four spinful NRs and eventually two symmetry-protected Weyl NRs. FM ferromagnetic, SOC spin-orbit coupling. The gray planes represent the mirror symmetry plane, which is parallel to the NR plane in the k space. With SOC, the two yellow NRs survive, while the two green NRs are gapped. The blue and red structures represent the energy surfaces above and below the energy of the NR, respectively. **b** Magnetic phase diagram (*H*−*T*) for EuGa_4_. SP spin-polarized phase, AFM antiferromagnetic phase. Inset shows the top and side views of the EuGa_4_ crystal structure. The empty circle symbols mark the magnetic phase boundary determined by magnetization measurements, see Supplementary Fig. 1 for the full *M*(*H*) data. **c**–**e** Band structures of EuGa_4_ in the paramagnetic (PM) phase without SOC, SP phase without SOC, and SP phase with magnetic moment along *c* axis with SOC, respectively. **c** The nodes circled in red (blue) represent the ones residing on (off) the mirror invariant planes. The vertical dashed lines mark the high-symmetry *k*-points. **d** Blue and red indicate two sets of spin-split bands. **e** The bands that host protected crossings are colored. **f** 3D view of the Weyl NRs from DFT calculations. Three pairs of NRs are shown in green, cyan, and red/blue, respectively. Note that small parts of the red/blue NRs near S on the *k*_*z*_ = ± 2*π*/*c* planes extend outside of the BZ. Symmetry operations fold these extended segments back to the *k*_*z*_ = 0 plane of the BZ. **g** Energy (*E*) surface of the bands that form the red/blue NRs. Blue and red indicate that the energy is above and below that of the NRs, respectively. **h** Top view of the red/blue Weyl NRs, with the color indicating the energy. Inset in **h** shows the zoom-in NR pair from the top of the panel. The legend is shown on the top.

Weyl NR states in a square lattice emerge as a result of spin degeneracy breaking and SOC, with mirror symmetry protection. Figure 1a illustrates this mechanism. Without SOC, square-net compounds with conduction bands derived from *p*_*x*_/*p*_*y*_ orbitals serve as a platform to host *spinless* four-fold degenerate diamond-shaped NRs in the mirror invariant plane (left, Fig. 1a)^28–31^. When ferromagnetism (FM) is introduced (middle, Fig. 1a), the spin degeneracy is lifted, resulting in four *spinful* NRs, with each NR two-fold degenerate. Finally, when SOC is turned on, only a subset of these spinful NRs survives, depending on the orientation of the magnetic moment *m*. When *m* is perpendicular to the mirror plane, the mirror symmetry is preserved. Therefore, one pair of NRs from bands with opposite mirror eigenvalues is protected, while the other pair of NRs with the same mirror eigenvalue is suppressed by opening band gaps. Since the spinless NRs in square-net materials typically have a small energy dispersion^28,30,31^, this mechanism offers an opportunity to create low-dispersion Weyl NR states.

EuGa_4_, which crystallizes in the BaAl_4_-type structure (space group *I*4/*mmm*)^32,33^ with Ga sublattice forming layered square nets (inset, Fig. 1b), proves suitable for realizing the Weyl NR states following this mechanism. Its magnetic phase diagram is shown in Fig. 1b. When *H* = 0 below *T*_N_ = 16.3 K, EuGa_4_ is an A-type antiferromagnet (AFM), with the Eu moments parallel to the *a* axis^34^. When **H** ∥ *c* is applied, the moments rotate towards the field direction until a phase transition to the spin-polarized (SP) state (or field-induced FM state^32^).

In the paramagnetic (PM) state above *T*_N_, there are three mirror reflection symmetries for the EuGa_4_ lattice: m_*z*_, m_*x*_ (or m_*y*_), and m_*xy*_, where the mirror planes are perpendicular to the *z*, *x* (or *y*), and the in-plane diagonal crystallographic axis, respectively. When the moments are ordered, at least two of these three mirror reflections are destroyed, depending on the specific magnetic configuration. When the magnetic moments are along the c axis (**m** ∥ *c*), the Eu layers act as the m_*z*_ mirror planes, which allows the formation of Weyl NR states. In Supplementary Note 3, we also provide an extended discussion on the mechanism of Weyl NR states in EuGa_4_ compared to Dirac/Weyl point states in the broad family of square-net topological semimetals.

The band structures of EuGa_4_ from DFT calculations in the PM state, the SP state without SOC, and the SP state with SOC are shown in Fig. 1c–e, respectively. In the PM state, the bands show multiple crossings, with the corresponding nodes divided into two groups, on (red circles) or off (blue circles) the mirror invariant planes at *k*_*z*_ = 0 and *k*_*z*_ = ± 2*π*/*c* (Fig. 1c). In the three-dimensional (3D) *k* space, these nodes, except the one along Γ − Z, extend to form lines (Supplementary Fig. 2). In particular, the NLs on the *k*_*z*_ = 0 and *k*_*z*_ = ± 2*π*/*c* planes exhibit NR geometry. When the spin is fully polarized in the Eu sublattice without SOC, two sets of spin-split bands form (Fig. 1d). When **m** ∥ *c* with SOC, only the crossings from bands with opposite mirror eigenvalues are retained (Fig. 1e), resulting in the formation of Weyl NRs, as shown in Fig. 1f. Depending on their band origins, these Weyl NRs can be categorized into three groups: the ones on the *k*_*z*_ = 0 plane (green), *k*_*z*_ = ± 2*π*/*c* planes (red/blue pair), and *k*_*z*_ = ± 2*π*/*c* planes (cyan). In particular, the red/blue NRs are found to sit very close to *E*_*F*_ with small energy variation of 0.18eV, although they span the whole *k*_*z*_ = ± 2*π*/*c* planes of the Brillouin zone (BZ) (Fig. 1f–h).

To experimentally validate the existence of the Weyl NR states, we provide below ARPES and QO measurements, which allow us to: (1) identify the spinless NR states in the PM state; and (2) determine the band splittings of these NRs in the SP state. When two pairs of spin-split bands cross in the mirror invariant plane, Weyl NR states are guaranteed.

### ARPES investigation of spinless NR states

**Fig. 2:**
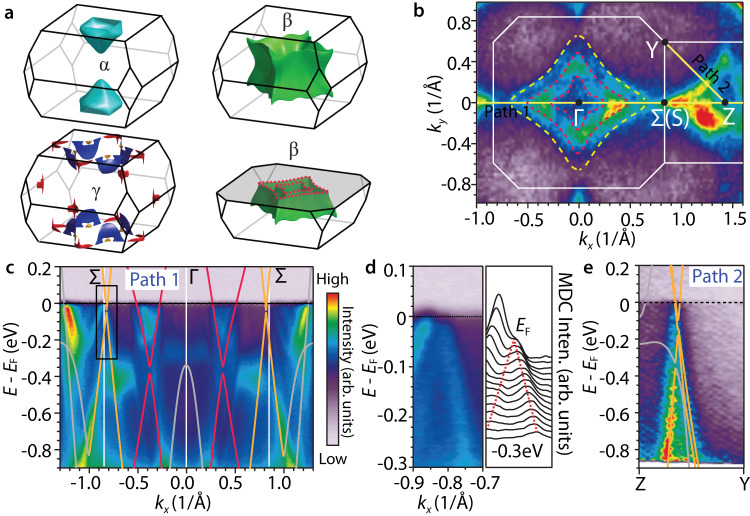
Electronic structure of EuGa_4_ in the PM phase. **a** Three groups of FS pockets: *α*, *β*, and *γ*, based on DFT calculations. The cross-sectional cut of the *β* pocket at the *k*_*z*_ = 0 plane is illustrated with dashed red lines. **b** ARPES measured FS with *hν* = 118 eV and *T* = 25 K. Two high-symmetry *k* − paths (yellow lines) are indicated for band dispersion analysis. The white lines mark the BZ boundary. The dashed red lines are the *k*_*z*_ = 0 cross sections of the *β* pocket, the same as those shown in **a**. The dashed yellow lines delineate the boundary within which the effect of *k*_*z*_-broadening is observed, as discussed in the text. **c** ARPES band dispersion along path 1 with *hν* = 120 eV. The solid lines are band structures from DFT calculations. Red and orange indicate the bands that form the NR1 and NR2, respectively, while the gray bands are irrelevant ones. Same applies to **e**. **d** Zoom-in view of the boxed region in **c**, with the MDC stacks shown on the right. **e**, ARPES band dispersion along the Z−Y path. The colorbar in **c** is shared for **d** and **e** as well.

As shown in Fig. 1c, there are two crossings along the Γ − Σ path; one is 0.36 eV below *E*_*F*_ and the other very close to *E*_*F*_. These two crossings and the one above *E*_*F*_ on the *k*_*z*_ = ± 2*π*/*c* plane extend to form three spinless NRs in the *k* space (denoted as NR1, NR2, and NR3, see Supplementary Fig. 2a). The Fermi surface (FS) pockets derived from these NR bands are accordingly divided into three groups: *α*, *β*, and *γ*, as shown in Fig. 2a.

Figure 2b shows the measured FS cross-section of EuGa_4_ at 25 K (PM phase), with a photon energy *hν* = 118 eV, which corresponds to the *k*_*z*_ ≈ 0 plane (for photon energy dependent data see Supplementary Fig. 3). Centered at the Γ point, the ARPES data show enhanced intensity within two concentric diamond rings (dashed red curves in Fig. 2b), which are exactly the inner and outer *k*_*z*_ = 0 cross sections of the *β* pocket from DFT calculations (Fig. 2a). Outside the outer diamond, finite ARPES intensity, albeit lower than the region in between, persists up to the dashed yellow boundary. The origin of its nonzero ARPES intensity is attributed to *k*_*z*_ broadening, considering the outward warping geometry of the *β* pocket along *k*_*z*_.

To view the band dispersion, we extracted the measured ARPES spectra along two high-symmetry paths, one along ${{\Sigma }}-{{\Gamma }}-{{\Sigma }}\left({{{{{{{\rm{S}}}}}}}}\right)-{{{{{{{\rm{Z}}}}}}}}$ (Fig. 2c), and the other along the diagonal Z − Y direction (Fig. 2e). For comparison, the DFT calculated band structure (lines) is overlaid on top, with red and orange indicating the NR1 and NR2 bands, respectively. Indeed, the nodes of the NR2 sit very close to *E*_*F*_, as is evident from the zoom-in band image and associated momentum distribution curves in Fig. 2d. Our data further show suppressed spectral weight near *E*_*F*_, suggesting the existence of a small gap. This is consistent with SOC induced gap (20 meV) at the crossing from DFT calculations. The ARPES spectra along the Z − Y path (Fig. 2e) also show clear linear-band crossings near *E*_*F*_, supporting the low dispersion feature along the ring for the NR2. As for the NR1, one branch of the bands appears to be clearer than the other (See the band dispersion along Γ − Σ in Supplementary Fig. 3b), possibly due to the matrix element effect.

Overall, the ARPES data supports the existence of spinless NR1 and NR2 in the PM phase of EuGa_4_, as predicted by theory. Particularly, the NR2 is confirmed to sit very close to *E*_*F*_ with small energy variation along the ring.

### Weyl NR states in the SP state

As shown in Fig. 1f, there are three pairs of Weyl NRs in the SP state of EuGa_4_. Consequently, there are three groups of FS pockets (Supplementary Fig. 5), which appear in pairs (one smaller and one larger) due to band splittings, although the shape is similar to that in the PM state (Fig. 2a). Quantum oscillations, which are a direct measure of the FS pockets, provide quantitative information about the band splitting and the energy of the Weyl NR states. The oscillation frequency *f* is related to the cross-sectional area *A*_*k*_ of the FS perpendicular to the applied magnetic field via the Onsager relation: *f* = (Φ_0_/2*π*^2^)*A*_*k*_, where Φ_0_ = 2.07 × 10^−15^ Tm^2^ is the flux quantum. By rotating the field, the QO frequency picks up an angle dependence, from which a 3D picture of the shape and size of the FS can be constructed.

**Fig. 3:**
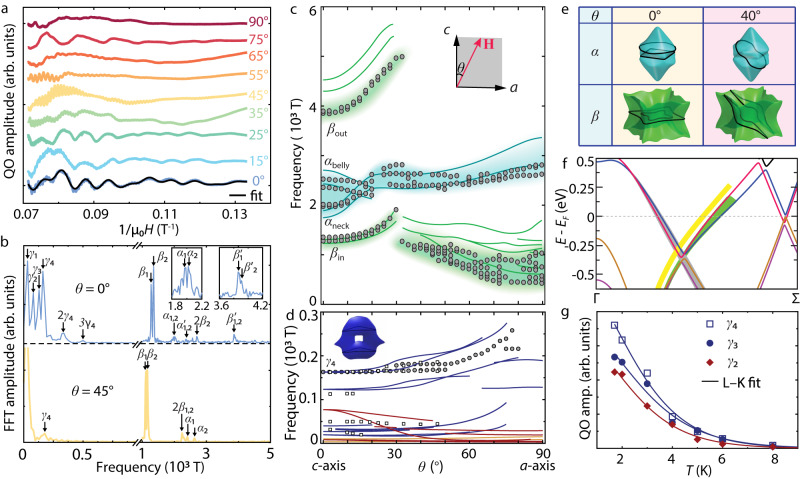
Fermi surface geometry of EuGa_4_ in SP phase from quantum oscillations. **a** A series of QO curves with *θ* ranging from 0^∘^ to 90^∘^. For the QO at 0^∘^, an L–K fit is shown. **b** Two representative FFT spectra of the QOs at *θ* = 0^∘^ and 45^∘^. The QO frequencies associated with the *α*, *β,* and *γ* pockets are labeled accordingly. The two insets in **c** show the zoom-in views of the FFT spectra near *α*_1_/*α*_2_ and ${\beta }_{1}^{{\prime} }$/${\beta }_{2}^{{\prime} }$ frequencies at *θ* = 0^∘^. **c**, **d** Angle dependent QO frequencies (circles) above and below 300 T, respectively. The QO frequencies shown in circles are measured using a lab magnetometer, while those shown in squares are determined by high-field measurements. The shadings in **c** act as a guide to the eyes. Inset in **c** illustrates the definition of rotation angle, *θ*. **H** indicates the applied magnetic field. The cyan and green lines in **c** are from theoretical predictions for the *α* and *β* pockets, respectively. The red, blue, and orange lines in **d** represent the theoretical prediction associated with the *γ* pockets of the same color as those illustrated in Fig. 2a. **d** Illustrates the extremal cyclotron orbits associated with the measured *γ*_4_ frequency at *θ* = 0^∘^. In Supplementary Fig. 7d, we illustrated all the extremal orbits with the *γ* pockets. **e** Illustration of the extremal orbits (black lines) of the *α* and *β* pockets, when *θ* = 0^∘^ and 40^∘^. **f** Band structure along Γ − Σ with the feedback from QO measurements. The energy of the gray-shaded bands is accurately described by theory based on the QO measurements, while that of the green-shaded band is underestimated by theory. The actual bands should have slightly higher energy, as indicated by the yellow shades. **g** Temperature dependent QO amplitude for the *γ*_4_, *γ*_3_, and *γ*_2_ oscillations at *θ* = 0^∘^ and their L–K fits (solid lines).

In Fig. 3a, we present a series of Shubnikov-de Haas (SdH) oscillations, with the field tilting from **H** ∥ *c* (*θ* = 0^∘^) towards **H** ∥ *a* (*θ* = 90^∘^). Figure 3b shows the fast Fourier transform (FFT) analysis of the QOs at two discrete angles: *θ* = 0^∘^ and 45^∘^. The contour plot of the FFT intensity at all measured angles is shown in the Supplementary Fig. 6 and the extracted QO frequencies are shown as circles in Fig. 3c, d.

At high frequencies (*f* > 300 T, Fig. 3c), the experimental data show good agreement with the theoretical prediction (colored lines) on the *α* and *β* pockets, with the colored shading as a guide. Starting at *θ* = 0^∘^, four pairs of QO frequencies (indicated by *β*_in_, *β*_out_, *α*_neck_, and *α*_belly_) are identified. The two close-lying frequencies within each pair have similar angular dependency, suggesting similar shape of FS and pointing to band splitting as their origin. As *θ* gradually increases from 0^∘^ to ~20^∘^, the *α*_neck_ and *α*_belly_ frequencies merge. By contrast, the *β*_in_ and *β*_out_ frequencies both increase with *θ*, until a sudden drop occurs at ~30^∘^. These features suggest a morphological change of extremal cyclotron orbits as the field rotates, which is the key to understanding the shape of probed FS pocket. The *α*_neck_ and *α*_belly_ frequencies at small *θ* arise because the *α* pockets have slight corrugations along the vertical axis, while the *β*_in_ and *β*_out_ frequencies are associated with the inner and outer cross-sectional areas of the torus-shaped *β* pockets (Fig. 3e). As *θ* increases beyond a certain critical angle (*θ*_c_ ~ 30^∘^), the extremal cross-section of the *β* pocket undergoes a change from the in-and-out to the sidewise pair.

With the shape of the *α* and *β* pockets determined, we now evaluate the energy of the bands, with a focus on the QO data at *θ* = 0^∘^ (**H** ∥ *c*). Figure 3f shows the DFT calculated band structure along the Γ − Σ path. The bands that give rise to *β*_in_ and *β*_out_ oscillations are marked with gray and green shadings, respectively. The excellent match between the experiment and theory on the *β*_in_ frequencies (Fig. 3c) indicates the accuracy of the *β*_in_ bands (gray shading, Fig. 3f) from DFT calculations. By comparison, the *β*_out_ frequency pair is ~400−600 T below the theoretical prediction, which means that the actual *β*_out_ bands (yellow shading) have slightly higher energy than the theoretical ones (green shading, Fig. 3f). Assuming a rigid band shift, the actual band energy is ~90−100 meV higher than the theoretical one. As for the *α*_neck_ and *α*_belly_ frequencies, the experimental ones are slightly higher and lower, respectively, than the theoretical predictions. This result suggests that the extent of neck-and-belly corrugation of the *α* pocket is less prominent than predicted by theory. Finally, based on the frequency difference of the *β*_in_, *β*_out_, *α*_neck_, and *α*_belly_ pairs, the energy of band splittings at *E*_*F*_ are determined to be 45, 10, 17, and 24 meV, respectively.

We now discuss the QO features of the *γ* pockets. According to the theoretical prediction, they are essentially composed of a series of side-by-side electron and hole pockets (Supplementary Fig. 5c) along the red/blue Weyl NRs. Since the energy of the nodes is very close to *E*_*F*_, these pockets are all small, giving rise to low-frequency QOs (Fig. 3d). FFT analysis of the measured QOs at *θ* = 0^∘^ reveals four frequency components: *γ*_1_ = 30 T, *γ*_2_ = 77 T, *γ*_3_ = 125 T, and *γ*_4_ = 163 T (Fig. 3b). A Lifshitz–Kosevich (L–K) fit (Fig. 3a) based on these four components reproduces well the measured QO curve. DFT calculations suggest that the blue pocket (inset, Fig. 3d) has the largest cross-sectional area at *θ* = 0^∘^. As *θ* increases, the predicted *f* remains nearly constant, and gradually bifurcates into two branches, eventually merging into one observable frequency at around 50^∘^ with weak increase with *θ*. Such subtle angle-dependent behavior is captured by the measured *γ*_4_ QO frequency, albeit with frequency values slightly smaller than the theoretical ones at high angles, as shown in Fig. 3d. Therefore, the *γ*_4_ frequency is identified as the signature of the blue pocket. Further high-field measurements reveal the existence of three smaller pockets (square symbols in Fig. 3d). However, the nature of these pockets is less obvious than the *γ*_4_ one, and more discussion is included in Supplementary Notes. 6 and 7. Due to the thermal broadening of chemical potential, the QO amplitude decreases with temperature, which provides a way to evaluate the effective mass and quantum mobility^35^. The measured QOs at different temperatures are shown in Supplementary Fig. 9. Based on the L–K fit to the temperature dependent QO amplitude (Fig. 3g), the effective masses of the *γ*_4_, *γ*_3_, and *γ*_2_ components are: ${m}^{*}\left({\gamma }_{4}\right)=0.74{m}_{e}$, ${m}^{*}\left({\gamma }_{3}\right)=0.68{m}_{e}$, and ${m}^{*}\left({\gamma }_{2}\right)=0.76{m}_{e}$, where *m*_*e*_ is the electron mass. These values are much higher than those in typical nonmagnetic TSMs, such as Cd_3_As_2_ (0.045*m*_*e*_)^36^ and NbP (0.076*m*_*e*_)^37^, and are also significantly higher than the DFT predictions (0.02*m*_*e*_ − 0.18*m*_*e*_) based on the single-particle picture (see Supplementary Note 8). The mass enhancement reflects the existence of electronic correlation effects in EuGa_4_. The quantum mobility at 1.7 K is estimated to be 830, 1180, and 1630 cm^2^/Vs for the *γ*_4_, *γ*_3_, and *γ*_2_ components, respectively, among the highest in all known magnetic TSMs. For comparison, the quantum mobility of the magnetic Weyl semimetal Co_3_Sn_2_S_2_ at 1.6 K is 106−221 cm^2^/Vs^38^, which is about one order of magnitude smaller than that in EuGa_4_.

Overall, with the identification of the spin-split bands for the *α*, *β,* and *γ* pockets, our QO data provide strong evidence for the existence of Weyl NR states in EuGa_4_, as predicted by theory. In particular, the red/blue NRs do cross *E*_*F*_ with small energy variation, giving rise to a series of small pockets, as revealed by the low-frequency QOs. In addition, the QO measurements reveal high quantum mobility.

### Electrical transport properties

**Fig. 4:**
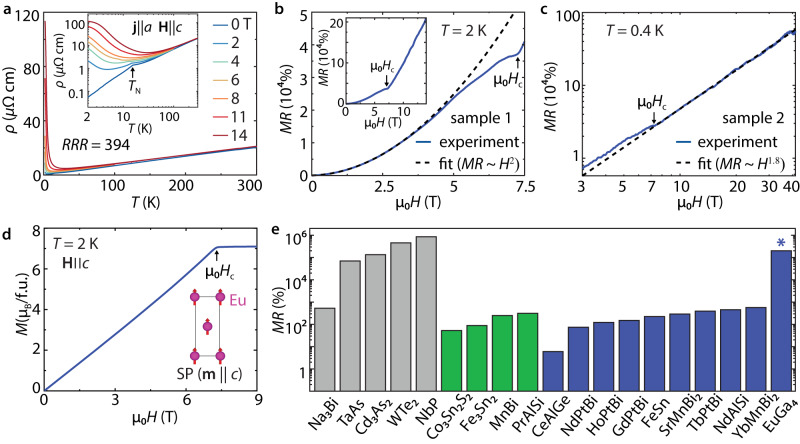
Large, non-saturating MR in EuGa_4_. **a** Temperature-dependent resistivity at selected fields. Inset shows the same data in a logarithmic scale, highlighting the low-*T* behaviors. The field is applied along the c axis (**H** ∥ *c*), while the current is along the a axis (**j** ∥ *a*). *RRR*, residual resistivity ratio; *T*_N_, Néel temperature. **b**, **c** The low- and high-field MR behaviors, respectively. The AFM-to-SP magnetic phase transition is indicated by the arrow. The *H*^2^ fit is performed on the MR curve from 0 to 3.5 T in **b**, while a power function fit is performed above *H*_*c*_ up to 41.5 T in **c**, with 1.8 as the exponent. Inset in **b** shows the MR curve up to 14 T. **d** Isothermal magnetization curves (**H** ∥ *c*) at *T* = 2 K. Fully spin-polarized phase is reached above *μ*_0_*H*_c_ = 7.4 T. Inset illustrates the SP state with the moments on Eu sublattices along the *c* axis. **e** Comparison of the measured MR in EuGa_4_ (marked by *) with other known TSMs. The nonmagnetic compounds are colored gray, while the ferromagnetic and antiferromagnetic ones are colored green and blue, respectively. See Supplementary Table 1 for the field, temperature, and reference information on the compounds in this plot.

The temperature-dependent resistivity $\rho \left(T\right)$ of EuGa_4_ in zero fields (Fig. 4a) reveals a typical metallic behavior, as *ρ* decreases monotonically with decreasing *T* down to 2 K. Below *T*_N_ = 16.3 K, the loss of spin disorder scattering induced a sharp drop, consistent with prior measurements^33,39^. The high residual resistivity ratio $RRR=\rho \left(300\,{{{{{{{\rm{K}}}}}}}}\right)/\rho \left(2\,{{{{{{{\rm{K}}}}}}}}\right)$ = 394 is indicative of high crystal quality. When **H** ∥ *c* is applied, the low-*T* resistivity exhibits an upturn in cooling. Such “turn-on” behavior by field suggests large MR response and high transport mobility, which have been seen in several representative nonmagnetic TSMs, such as TaAs^40^ and NbP^37^.

We are interested in the field dependence of the MR response. Qualitatively different field dependence is observed below and above *μ*_0_*H*_*c*_ = 7.4 T (Fig. 4b, c, *μ*_0_ is the vacuum permeability), which marks the magnetic phase transition at 2 K in EuGa_4_. The MR response in the AFM phase can be well described by an *H*^2^ dependence below ~3.5 T, and levels off as *μ*_0_*H* increases further towards *μ*_0_*H*_*c*_ (Fig. 4b). This is a typical behavior seen in uncompensated semimetals^41^. However, once the system enters the SP phase (*H* > *H*_*c*_), where the Weyl NR states are hosted, the MR shows an abrupt upturn (Fig. 4b inset) and continues to increase without any signs of saturation up to ~40 T (Fig. 4c and Supplementary Fig. 10).

In AFM metals, field-induced spin fluctuations can be a cause for an increase in resistivity. However, as the field continuously increases in the fully SP state, the electron scattering caused by spin fluctuations is increasingly suppressed, which should lead to a resistivity decrease. This appears in quite a few AFM metals, such as MnBi_2_Te_4_^42^ and EuPtSi^43^, but is not the case in EuGa_4_. Furthermore, the carrier compensation mechanism for the non-saturating MR demands a perfect balance of electron and hole carrier density, *n*_*e*_ = *n*_*h*_; a slight deviation from this condition will lead to a saturating MR at high field^44^. This is the case in Bi, where the MR deviates from the power-law scaling at ~6 T, and reaches full saturation at ~30 T^45^. Given the carrier density in EuGa_4_ does not meet the carrier compensation condition (see Supplementary Note 11 for the estimate of the carrier density), a different mechanism is expected to explain the non-saturating MR behavior.

We investigated the magnetotransport properties of a Weyl nodal ring semimetal with both semi-classical and fully quantum mechanical approaches (see Supplementary Note 12 for more details on the theoretical modellings and discussions). We find that non-saturating MR naturally arises in the Weyl nodal ring system without the requirement of perfect electron-and-hole carrier compensation. This unusual behavior benefits from the negligibly small Hall conductivity, which occurs due to the sign reversal of the Fermi velocity across the nodal ring. Notably, our theoretical model also predicts sub-quadratic power-law scaling for MR, which resonates with the experimental observations (Fig. 4c). Here we further note that this mechanism is different from the quantum magnetoresistance proposed by Abrikosov^46^, where linear and non-saturating MR is achieved only when electrons are forced to occupy the lowest Landau level (quantum limit) in a linear-band system. Since most of the conducting carriers come from the large *α*- and *β-*pockets in EuGa_4_, the Abrikosov mechanism is not expected to play a dominant role in the non-saturating MR behavior.

When comparing the MR in EuGa_4_ with the values in other known magnetic TSMs (Fig. 4e), EuGa_4_ stands out. The MR at 2 K and 14 T exceeds 2 × 10^5^%, which is more than two orders of magnitude larger than those in other known magnetic TSMs (green and blue), and even comparable to that in the nonmagnetic ones (gray). As the field increases up to ~40 T, a non-saturating MR ~5 × 10^5^ % is observed in EuGa_4_ (Fig. 4c).

We emphasize that the AFM state of EuGa_4_ with or without moment canting would fail to provide the required symmetry protection for the existence of Weyl NR states. In the AFM ground state, the two magnetic sublattices (**m** ∥ *a*) are connected by the joint translation and time-reversal symmetry {T∣(1/2, 1/2, 1/2)}. Consequently, the spin degeneracy is not lifted and no Weyl NR states are supported in the presence of SOC. In the spin-canted state, the {T∣(1/2, 1/2, 1/2)} symmetry is broken along with spin splitting. However, since the spin canting breaks all the mirror symmetries: m_*x*_, m_*xy*_, and m_*z*_, no Weyl NRs should exist either. Only when the system reaches the SP state above *H*_*c*_ and the mirror symmetry m_*z*_ is recovered, do the Weyl NR states appear. Based on this symmetry analysis, we conclude that a field-induced topological phase transition occurs in EuGa_4_ along with the AFM-SP magnetic transition. The upturn sub-quadratic MR increase is related to this transition.

## Discussion

We present the magnetic square-net compound EuGa_4_ as a host for Weyl NR states. Our combined ARPES and QO measurements provide strong evidence for the existence of Weyl NRs close to the Fermi level in the SP phase, consistent with our DFT predictions. In particular, one pair of Weyl NRs is found to cross *E*_*F*_, with a small energy variation of 165−195 meV although it spans the whole plane of the BZ. With high carrier mobility in EuGa_4_, we reveal clear features of Landau quantization of these NR states. Arguably, the most interesting feature is the qualitatively different field-dependent MR behaviors in the AFM and SP phase, where the Weyl NR states are stabilized only in the latter. While the MR curves in the AFM phase gradually level off, they pick up a fast upturn increase without any sign of saturation up to ~40 T in the SP phase. These behaviors cannot be attributed to a carrier compensation mechanism. Instead, we developed a theoretical model that naturally explains the non-saturating MR, highlighting the role of the Weyl nodal ring state. At 14 T and 2 K, the measured MR exceeds 2 × 10^5^%, more than two orders of magnitude larger than those in other known magnetic TSMs. Our work thus provides insight for the design of magnetic materials with large MR.

## Methods

### Sample growth and characterization

Single crystals of EuGa_4_ were grown in an excess of gallium (Ga) via a self-flux technique. Europium (Eu) and Ga were mixed in a ratio of 1:9 then placed in an alumna crucible and evacuated in a quartz ampule. The mix was heated to 900 ^∘^C over 2 hours and subsequently slowly cooled over a period of 60 hours down to 700 ^∘^C, after which the crystals were separated from the excess liquid flux using a centrifuge. EuGa_4_ forms plate-like crystals with the biggest surface area corresponding to the crystallographic *a*–*b* plane. The largest crystals have lateral sizes up to 5mm. The single crystals were confirmed to have BaAl_4_ type of structure with powder x-ray diffraction. Rietveld structural refinement was achieved and fit to the measured intensities. We extracted the structural parameters for EuGa_4_, listed in Supplementary Table 3, which served as the input for the density functional theory calculations.

### Angle-resolved photoemission spectroscopy experiments

ARPES experiments were carried out at Beamline 5-2 of the Stanford Synchrotron Radiation Lightsource (SSRL), Beamline 4.0.3, and Beamline 7.0.2 (MAESTRO) of the Advanced Light Source. EuGa_4_ samples were cleaved in situ to expose the (001) surface in an ultrahigh vacuum chamber with base pressure 3 × 10^−11^ Torr. The ARPES data were acquired within 5 hours after cleaving to minimize the effects of surface degradation. The lateral size of the beam is smaller than 50 × 50 μm^2^. Fermi surfaces and energy-momentum dispersions in Fig. 2 were collected at 118 and 120 eV, covering the entire Brillouin zone. The photon energy-dependent data along $\overline{{{\Sigma }}}$-$\overline{{{\Gamma }}}$-$\overline{{{\Sigma }}}$ path were taken with photon energies ranging from 60 to 180 eV.

### Electrical transport and SdH oscillation measurements

The electrical transport and SdH quantum oscillation experiments were carried out in a standard four-probe geometry in a lab magnetometer, Quantum Design DynaCool system, with a field up to 14 T. The high-field measurements were performed at the National High Magnetic Field Laboratory at Tallahassee, with fields up to 41.5 T.

The angular-dependent QO measurements in the Dynacool system were performed by rotating the sample in the a-c plane from **H** ∥ *c* to **H** ∥ *a*, with the current along b (**j** ∥ *b*). When **H** ∥ *c*, we measured QOs at different temperatures to evaluate the cyclotron effective mass. The oscillations were obtained after subtracting a polynomial background from the field-dependent resistivity data, after which they were analyzed with a FFT as a function of inverse field. The angle-dependent QO frequencies from the *α*, *β,* and *γ* pockets were extracted to compare with the theory. The frequency resolution/error is largely determined by the measured field window and sampling frequency. For the measurement using the lab magnetometer, the resolution of the QO frequency is approaching 20 T.

To evaluate the cyclotron effective mass (m^*^) and estimate the quantum lifetime (*τ*_*q*_) of the *γ* FS pockets, we performed L–K fits to the measurements with four frequency components. Each QO component is described by:

$${{\Delta }}\rho \propto \frac{\lambda T}{\sinh (\lambda T)}{e}^{-\lambda T}\cos \left[2\pi \left(\frac{f}{B}-\frac{1}{2}+\beta+\delta \right)\right]$$

where *λ* = (2*π*^2^*k*_*B*_m^*^)/(*ℏeB*). *ℏ* and *k*_B_ are the reduced Plank’s constant and the Boltzmann constant, respectively. *T*_D_ is the Dingle temperature, *f* is the QO frequency, 2*πβ* is the Berry phase and *δ* is a phase shift factor. The quantum lifetime (*τ*_*q*_) and mobility (*μ*_*q*_) were calculated by *τ*_*q*_ = *ℏ*/2*πk*_B_*T*_D_ and *μ*_*q*_ = *eτ*_*q*_/m^*^.

### Density-functional calculations

DFT calculations were performed by using the code of Vienna ab-initio simulation package^47^, with the experimental lattice parameters and atomic positions (Supplementary Table 3) as the input. To account for the localized f-electrons, an on-site Hubbard *U* = 5 eV was applied on Eu-4f orbitals^48^. The calculated magnetic moment is ~6.9 *μ*_B_/Eu, close to the experimentally measured one. The DFT electronic band structure and magnetization were double-checked by the full-potential local-orbital code with localized atomic basis and full potential^49^. To calculate the Fermi surfaces, we projected Bloch wavefunctions onto maximally localized Wannier functions (MLWFs)^50^, and tight-binding model Hamiltonians were constructed from the MLWFs overlap matrix. By performing constant energy contour slices, we were able to obtain the extremal cross-sectional area which is related to the frequency of each pocket as a function of angle and can be used to compare with the quantum oscillation measurements.

### Supplementary information

Supplementary Information

Peer Review File

## Data Availability

All data are available in the main text or the supplementary information files. All raw data generated during the current study are available from the corresponding authors upon request.

## Code availability

The code for the theoretical calculations during the current study are available from the corresponding authors upon request.
