# Supplementary material for: Weyl nodal ring states and Landau quantization with very large magnetoresistance in square-net magnet EuGa4
Source: Nat Commun. 2023 Sep 19;14:5812. doi: 10.1038/s41467-023-40767-z (PMC10509256; doi:10.1038/s41467-023-40767-z)
Supplement: Supplementary file 1 — Supplementary Information [file 41467_2023_40767_MOESM1_ESM.pdf]

Supplementary Information for Weyl nodal ring states and Landau quantization with very large magnetoresistance in square-net magnet $EuGa_4$

Shiming Lei[*,#], Kevin Allen[*], Jianwei Huang, Jaime M. Moya, Tsz Chun Wu, Brian Casas, Yichen Zhang, Ji Seop Oh, Makoto Hashimoto, Donghui Lu, Jonathan Denlinger, Chris Jozwiak, Aaron Bostwick, Eli Rotenberg, Luis Balicas, Robert Birgeneau, Matthew S. Foster, Ming Yi, Yan Sun[#], Emilia Morosan[#]

[*]These authors contributed equally to this work.

[#]Correspondence to: phslei@ust.hk, sunyan@imr.ac.cn, emorosan@rice.edu

This supplementary information includes:

Supplementary note 1: Magnetic phase diagram of $EuGa_4$
Supplementary note 2: Spinless nodal lines in the paramagnetic $EuGa_4$
Supplementary note 3: Weyl NR state and Dirac/Weyl point states in square-net materials
Supplementary note 4: Photon energy dependent data and more ARPES spectra
Supplementary note 5: DFT predicted Fermi surface of $EuGa_4$ in the SP phase
Supplementary note 6: Quantum oscillation measurements
Supplementary note 7: γ pockets in the SP phase and their extremal cross-sectional orbits
Supplementary note 8: Cyclotron effective mass of the γ pockets
Supplementary note 9: Large, non-saturating MR in $EuGa_4$
Supplementary note 10: MR value of $EuGa_4$ compared to that of other known TSMs
Supplementary note 11: Carrier density of $EuGa_4$
Supplementary note 12: Magnetotransport theory of Weyl nodal-ring semimetals
Supplementary note 13: Structural refinement from powder x-ray diffraction

Supplementary Note 1. Magnetic phase diagram of $EuGa_4$

Supplementary Figure 1a shows the $H - T$ phase diagram determined from isothermal magnetization measurements. The measured $M(H)$ curves at different temperatures are presented in Supplementary Fig. 1b. The magnetic phase boundary (dots in Supplementary Fig. 1a) is determined from the derivative dM/dH, as exemplified in Supplementary Fig. 1c (dashed line, right axis). Note that the magnetic moment saturates to 7 μ_B/Eu in the spin-polarized (SP) phase above $\mu_0 H = 7.4\,\mathrm{T}$ at $2\,\mathrm{K}$.

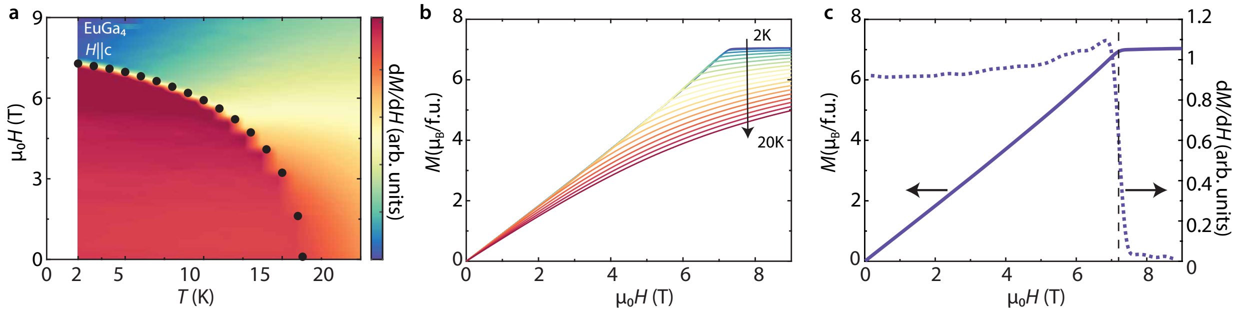

Supplementary Figure 1: **a**, $H - T$ magnetic phase diagram for $EuGa_4$ with magnetic field $H \parallel c$, where the contour plot represents dM/dH values. **b**, ***M*(*H*)** curves measured with temperatures from $2\,\mathrm{K}$ to $20\,\mathrm{K}$ up to $\mu_0 H = 9\,\mathrm{T}$. **c**, Field dependent magnetization at $2\,\mathrm{K}$ along with the dM/dH curve where the dashed vertical line indicates the critical field for magnetic phase transition.

Supplementary Note 2. Spinless nodal lines in the paramagnetic $EuGa_4$

Supplementary Figure 2a shows the spinless NLs in the paramagnetic (PM) state for $EuGa_4$, in the absence of spin-orbit-coupling (SOC). The NLs in the $k_z = 0$ and $k_z = \pm 2\pi/c$ planes all form closed NRs (indicated by NR1, NR2, and NR3). Along the $\Gamma - \Sigma$ path, there are two crossings below E_F (circled in Supplementary Fig. 2b), as discussed in the main text. These two nodes extend to form two NRs (NR1 and NR2) in the 3D k space. Note that only small parts of the NR2 on the $k_z = \pm 2\pi/c$ planes reside outside of the BZ. However, for this specific type of BZ which is associated with the body centered tetragonal cell, symmetry dictates that the electronic structure on the $k_z = \pm 2\pi/c$ planes in the neighboring BZ is the same as that on the $k_z = 0$ plane in the original BZ. Therefore, the majority part of the NR2 lives on the $k_z = \pm 2\pi/c$ planes of the original BZ, while the other parts are folded onto the $k_z = 0$ plane by symmetry. ARPES spectra along the $\Gamma - \Sigma$ path are able to access the information on the nodes from both NR1 and NR2.

Compared with the NR1 and NR2, the energy of NR3 is above E_F. Interestingly, the NR3

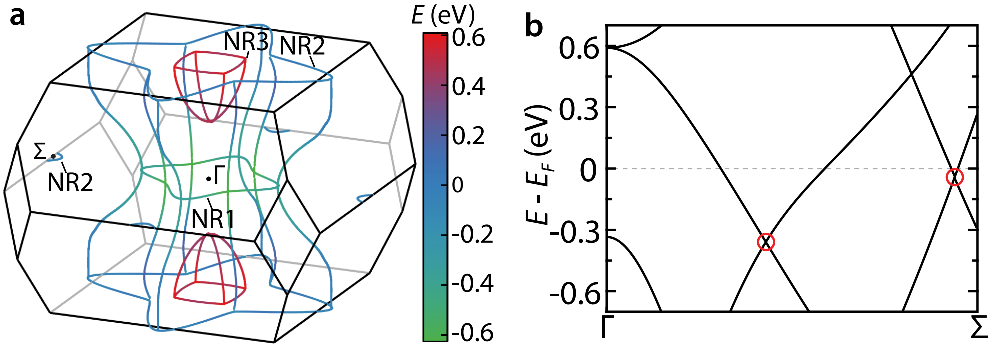

Supplementary Figure 2: The spinless NL state in paramagnetic state of $EuGa_4$. **a**, The NL network formed by the spinless NLs based on DFT calculations. SOC is not included. Color maps the energy of the NLs. Legend is shown on the right. The NLs in the $kz = 0$ and $kz = \pm 2\pi/c$ planes all form a closed NR geometry, with small energy dispersion along the ring. NR1, NR2, and NR3 denote three different NRs. **b**, The band structure along the $\Gamma - \Sigma$ path. The two nodes below E_F are circled. They extend to form the NR1 and NR2 in the 3D k-space.

connects two additional NRs that extend in the $k_x = \pm k_y$ planes, forming a cage-shaped network. The *topological nodal chains* with two NLs touching were first proposed in non-symmorphic crystals [1], but were also investigated later in a theoretical work on symmorphic crystals [2]. The bands that form the NR1, NR2 and NR3 lead to the formation of three groups of Fermi surface (FS) pockets, as shown in Fig. **2a** in the main text. These NR bands in the PM state undergo band splittings in the spin-polarized (SP) state, resulting in the formation of the three pairs of Weyl NRs.

Supplementary Note 3. Weyl NR and Dirac/Weyl point states in square-net materials

Square-net compounds with conduction bands derived from p_x/p_y orbitals are known to be a material platform to host the Dirac nodal lines (four-fold degenerate considering the spin degree of freedom) in the absence of SOC [3, 4]. Among the square-net topological semimetals, materials with the formula of MXZ and MXZ_2 are most heavily studied, as discussed in the recent review article [4]. To date, the focus of these studies has been primarily on the Dirac and Weyl point states and their associated physical properties. $SrMnBi_2$ [5, 6] and $YbMnBi_2$ [7] are two exemplary MXZ_2 compounds that were studied. The former one (antiferromagnetic ground state [8]) features anisotropic Dirac band dispersions. However, SOC opens a small gap of ~ 40 meV at the Dirac point [5]. For the latter, the spin-degeneracy can be lifted by the spin canting in the canted antiferromagnetic phase, and the band structure calculation points to the realization of Weyl point state [7]. Compared to the Dirac/Weyl point states studied in these earlier works, here we aim to

establish a different topological semimetal state, Weyl nodal line, where the spinful conduction and valence bands cross along curves in momentum space, rather than at discrete points [9, 10]. In addition, the Weyl NR state in $EuGa_4$ is robust against SOC.

Supplementary Note 4. Photon energy dependent ARPES data and more ARPES spectra

The FS cross section in the $k_y - k_z$ plane is measured by varying the photon energies from 60 to $180\,\mathrm{eV}$, as shown in Supplementary Fig. 3a. The band dispersion measured with the photon energy of $120\,\mathrm{eV}$ corresponds to the $k_z = 0$ plane.

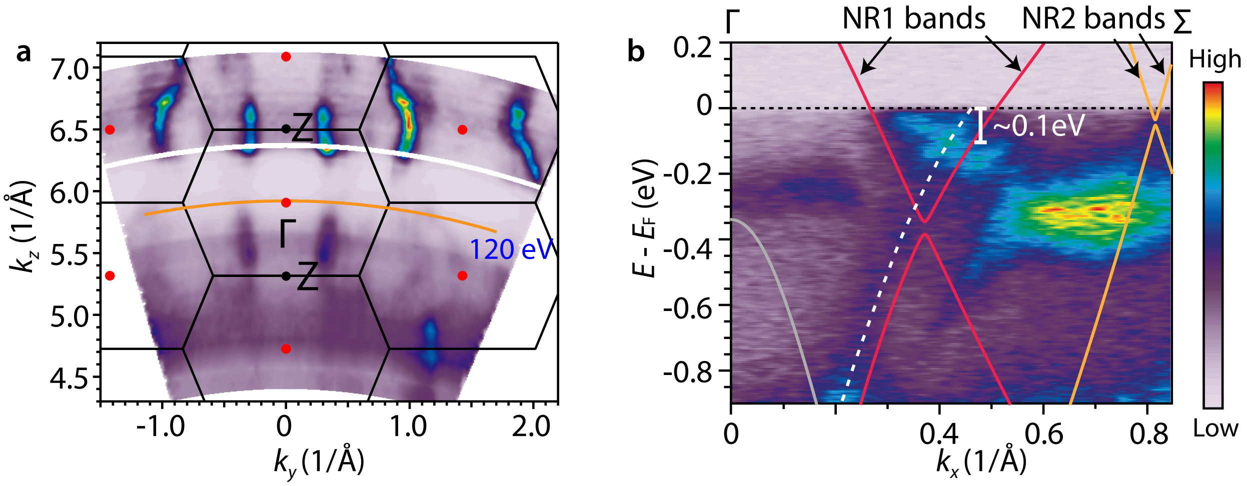

Supplementary Figure 3: **a**, k_z dependent ARPES intensity along the $\bar{\Sigma}$-$\bar{\Gamma}$-$\bar{\Sigma}$ path, measured with varying photon energy. The band dispersion along the $\Sigma - \Gamma - \Sigma\,(\mathrm{S}) - \mathrm{Z}$ path shown in Fig. 2c in the text is measured with photon energy of $h\nu = 120\,\mathrm{eV}$. **b**, Band image along $\Gamma - \Sigma$. The dashed white line delineates a measured band, which is about $0.1\,\mathrm{eV}$ higher than the corresponding branch of the NR1 bands from DFT calculations (red lines). Yellow lines indicate the NR2 bands from DFT calculations.

To confirm the reproducibility of the ARPES spectra, we repeated the ARPES measurements on a different $EuGa_4$ single crystal. The measured ARPES FS at the $k_z = 2\pi/c$ plane is shown in Supplementary Fig. 4a, featuring the spinless Dirac NR2, consistent with the DFT prediction (see the illustration in Supplementary Fig. 2a). We also checked the temperature dependent ARPES spectra to evaluate any observable change in the electronic structure from PM to AFM phase. Supplementary Figures 4b,d show the ARPES spectra measured at 10 K (AFM phase) and 30 K (PM phase). Unfortunately, we are not able to identify any change in the electronic structure. We also measured the temperature dependent momentum distribution curves (MDCs) and plot

its constant energy contour at E_F in Supplementary Fig. 4c; no clear changes can be identified across the AFM transition either. Future high-resolution ARPES experiments, such as laser-ARPES, focusing on the electronic structure around E_F would be crucial in revealing the subtle electronic structure change resulting from the AFM order.

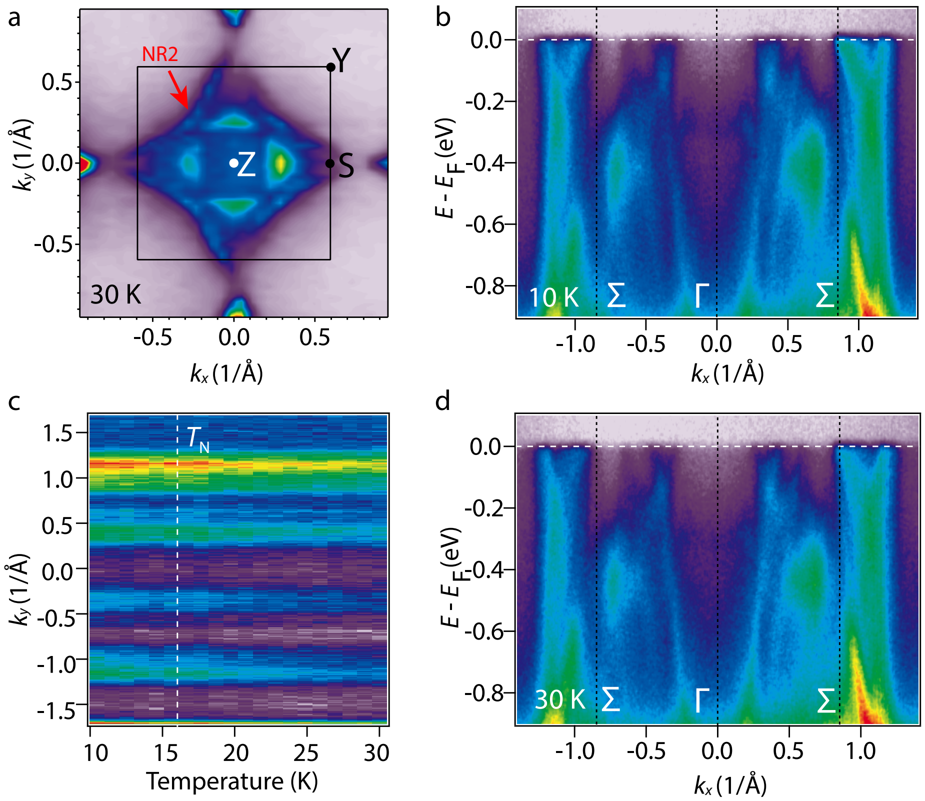

Supplementary Figure 4: **a**, ARPES measured FS cross section at the $k_z = 2\pi/c$ plane with $h\nu = 146$ eV and $T = 30$ K on a different crystal, featuring the spinless NR2 crossing the Brillouin zone, which is consistent with the DFT prediction (Supplementary Fig. 2a). **b,d,** ARPES band dispersion along the $\Sigma - \Gamma - \Sigma$ path measured at 10 K and 30 K, respectively. **c,** Constant energy contour of the MDC curves along the $\Sigma - \Gamma - \Sigma$ path at E_F, measured at a series of different temperatures ranging from 10 K to 30 K. The dashed line marks the Néel temperature, T_N.

Supplementary Note 5. DFT predicted Fermi surface of $EuGa_4$ in the SP phase

The bands that form the NR1, NR2, and NR3 (Supplementary Fig. 2a) in the PM phase of $EuGa_4$ undergo band splittings in the SP phase, which lead to the formation of three groups of FS pockets, as shown in Supplementary Fig. 5. Each group of FS pockets appear in pairs.

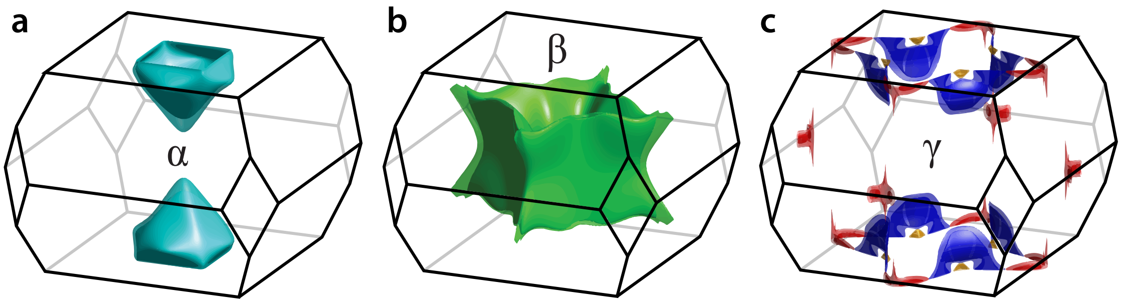

Supplementary Figure 5: Three groups of Fermi surface pockets of $EuGa_4$ in the SP phase.

Supplementary Note 6. Quantum oscillation measurements

SdH oscillations measured in our lab magnetometer (up to $14\,\mathrm{T}$) at various angles are sampled with a small angle increment of $2.5°$ from $0°$ to $90°$, as shown in Supplementary Fig. 6a. The contour plot of the fast Fourier transform (FFT) intensity is shown in Supplementary Fig. 6b. The peak locations are then extracted and marked as circles, as shown in Supplementary Fig. 6c-e. In addition to the measurements in a lab magnetometer, we have also performed SdH measurements on a separate sample in high field facilities up to $41.5\,\mathrm{T}$, but with bigger angle increment. The contour plot of the FFT intensity is shown in Supplementary Fig. 6f. Note that the measurement geometries are slightly different between the high field and lab magnetometer measurements. For the former, the current is applied along the a-axis ($j \parallel a$), while the field is rotating in the $a - c$ plane of the sample. For the latter, the current is applied along the b-axis ($j \parallel b$), which is always perpendicular to the field rotation plane ($a - c$ plane of the sample). In the high-field measurement, the MR response is significantly reduced when the field is rotated to approach the current direction. The data become noisy when the rotation angle is larger than $55°$, making it difficult to extract the FFT peaks. Nevertheless, two clear trends of QO frequency evolution can be identified up to $\sim 50°$ and two other trends up to $\sim 15°$, as marked by the square symbols in Supplementary Fig. 6f. QO frequencies with low FFT amplitude and broadened peaks are not labeled due to the difficulty of identifying the precise peak locations. QO frequencies below $\sim 20\,\mathrm{T}$ are not labeled either due to the limited resolution of measurements.

With the lab magnetometer measurements, we identified the angular evolution of QO frequencies for the α and β pockets. Combining the results from the lab magnetometer and the high-field measurements, we identified the evolution of the γ_4 frequency, which is consistent with the predicted features based on the outer blue γ pocket shown in Supplementary Fig. 5c. In addition to the γ_4 frequency, both lab magnetometer and the high-field SdH measurements reveal multiple smaller frequencies. Based on the DFT calculations, there are indeed multiple extremal cyclotron orbits

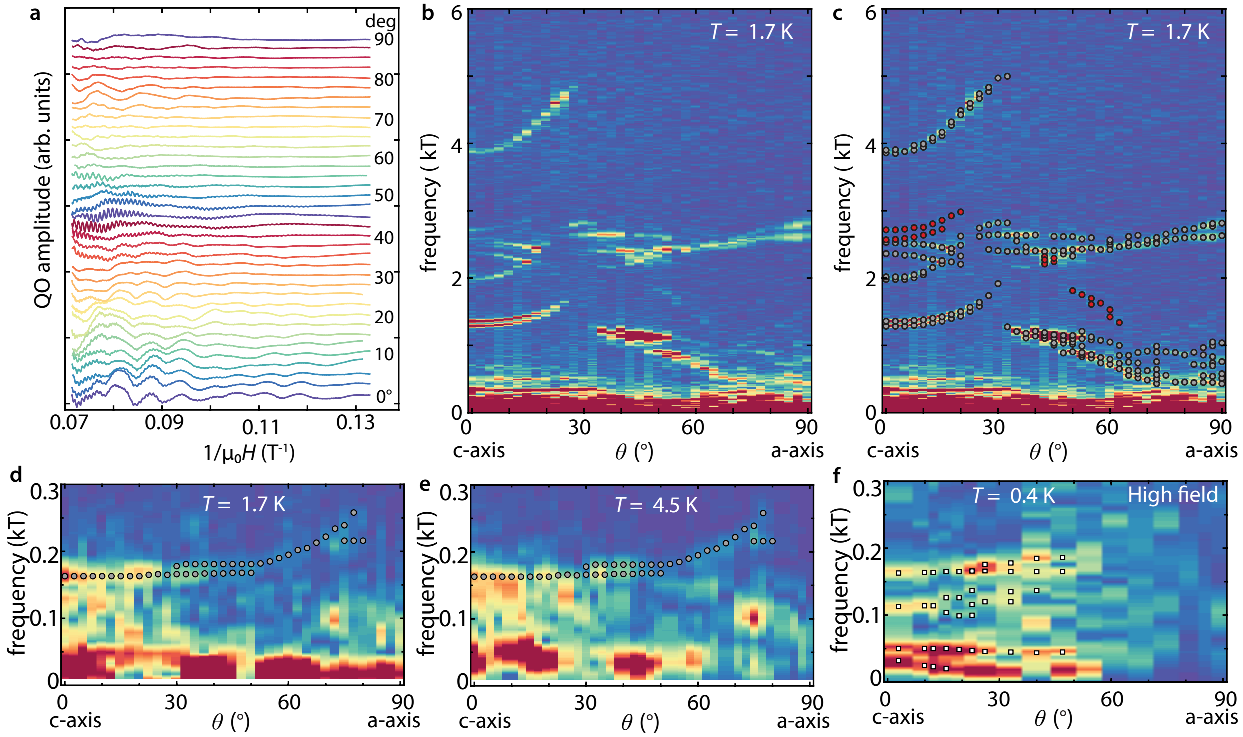

Supplementary Figure 6: Angle dependent SdH oscillations at $T = 1.7\,\mathrm{K}$, in the SP phase of $\mathrm{EuGa_4}$. **a,** Quantum oscillation after background subtraction at various angles from $0°$ to $90°$. The curves are purposely vertically shifted for better visualization. **b,c,** Contour plot of the FFT intensity of the QOs at each angle, and the same plot with peak locations marked by the circles. Note that the red circles indicate the harmonic frequency. **d-f,** Contour plots of the FFT intensity of the QOs at low frequencies. Note that panel (**d**) is the zoom-in view of the low-frequency region of panel (**b**). Panel (**e**) is measured at $T = 4.5\,\mathrm{K}$. Panel (**f**) is the one measured with fields up to $41.5\,\mathrm{T}$ at $T = 0.4\,\mathrm{K}$.

for the γ pockets (see Supplementary Fig. 7), as we will discuss in the next section. The resulting QOs frequencies are packed in a small window. Additionally, there are harmonics and magnetic breakdown with these low-frequency QOs. These factors make the correct identification of the origin of these lower-frequency QOs more challenging. A further investigation up to higher fields and with a finer step size will help.

We notice that de Haas-van Alphen oscillation measurements were performed to probe the Fermi surface geometry in $\mathrm{EuGa_4}$ in a prior study [11]. Unfortunately, the QOs with frequencies $f > 2500\,\mathrm{T}$ were not resolved when $H \parallel c$. Therefore, this work was not able to identify the α_{belly} and β_{out} frequencies. Furthermore, the small γ pockets and their QO features were not resolved or

discussed. The topological characters of the bands that lead to the formation of these pockets were unknown.

Supplementary Note 7. γ pockets in the SP phase and their extremal cross-sectional orbits

Based on the ARPES measurements on the PM $EuGa_4$, we have concluded that one branch of the bands that lead to the formation of the β pockets has slightly higher energy ($\sim 0.1\,$eV) than the theoretical prediction (see Supplementary Fig. 3b). Consequently, the outer cross section of the β pockets should have smaller area than the theoretical value. This conclusion is consistent with our QO measurements in the SP phase. The measured β_{out} QO frequencies are $400 - 600$ T below the theoretical predictions. Assuming a rigid band correction, an upshift of the theoretical bands by $\sim 90 - 100$ meV (illustrated in Fig. 3f in the main text) is required to reproduce the measured β_{out} QO frequencies.

The knowledge of the upshift energy correction to the β_{out} bands is helpful for the determination of the energy correction to the DFT bands that form the γ pockets, since they partially share the same band characters. In particular, the blue hole (red electron) pockets (see the illustration in Supplementary Fig. 5c) will be larger (smaller) than the theoretical predictions, along with the upshift of the β_{out} bands. However, the exact value of the energy shift of the bands that form the γ pockets can be smaller or larger than $90 - 100$ meV, due to the existence of k_z dispersion. One simple treatment is to rigidly adjust the Fermi energy (E_F) of all the pertinent bands that are responsible for the formation of the probed pockets. In reality, however, it is likely that the pockets are formed by two or more bands, but only one of them needs an adjustment while the others do not [12]. In this scenario, an E_F adjustment to all the bands by one common number can be considered as an averaging treatment.

We have evaluated the E_F adjustment to the bands that form the γ pockets. In particular, we find that an upshift of the bands, or equivalently a lowering of E_F by $35\,$meV is necessary to reproduce the measured QO frequency of $163\,$T ($\theta = 0°$) from the blue pockets (Supplementary Fig. 7). The required upshift of the bands is consistent with the expectation based on the ARPES results. As for the red pockets (Supplementary Fig. 7), our ARPES measurements suggest that the band crossing near the Σ point in the BZ is $20 \pm 10\,$meV higher than the theoretical prediction. Therefore, the E_F of the bands is lowered by $20\,$meV to obtain the theoretical QO frequencies associated with the red pockets (Supplementary Fig. 7). Overall, our QO measurements indicate that a small upshift of the DFT bands by $20 - 35\,$meV is necessary to understand the γ pockets.

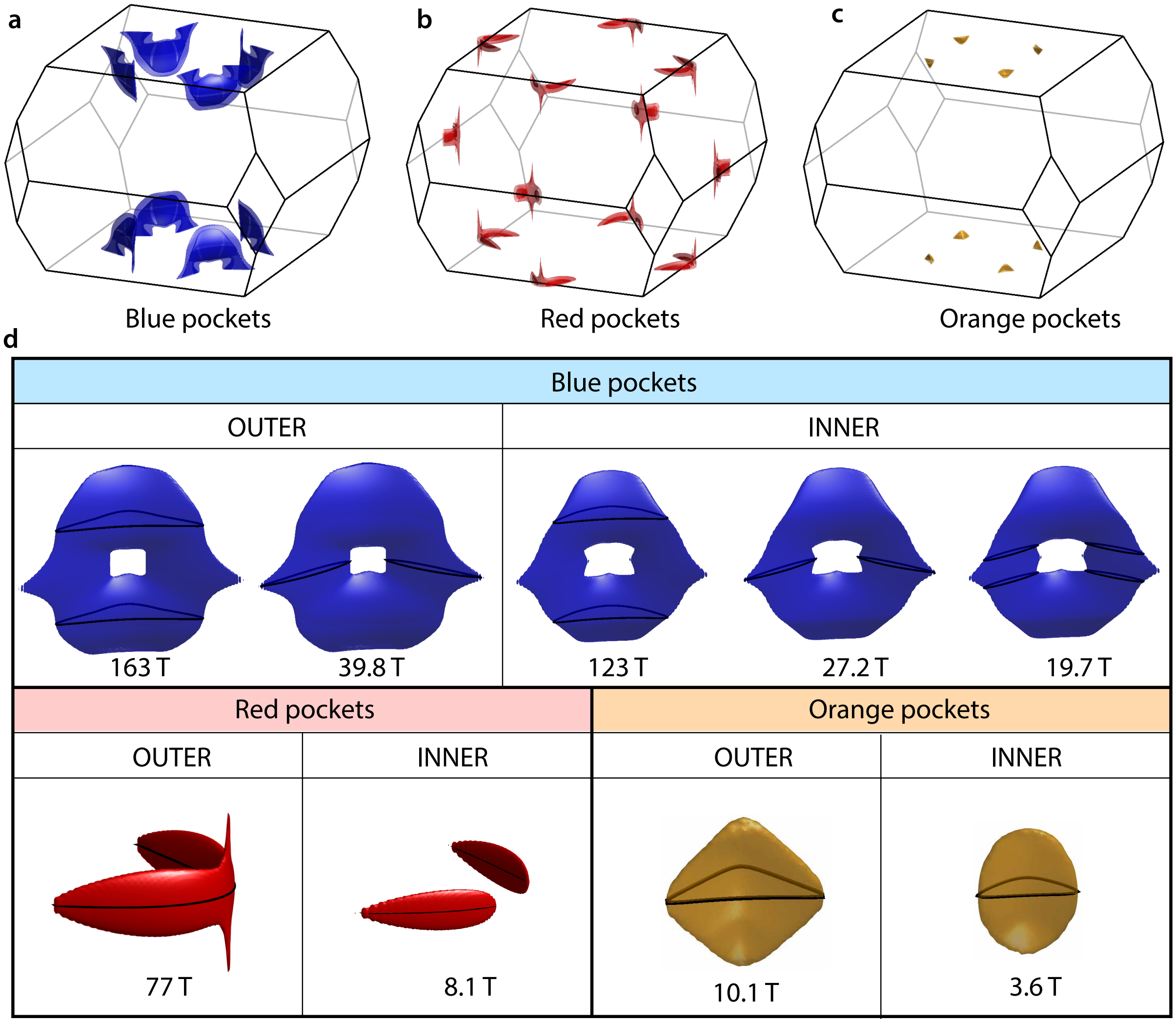

Supplementary Figure 7: Fermi surface pockets of the γ pockets. **a-c,** The blue, red, and orange pockets in the $EuGa_4$ Brillouin zone. Note that the outer pockets are rendered semi-transparent so that the inner ones are revealed. **d,** Enlarged view of each individual blue, red, and orange pocket. The black lines illustrate the extremal cross-sectional orbits of each pocket when $H \parallel c$. The number below each pocket indicates the QO frequency of the orbit in the unit of Tesla.

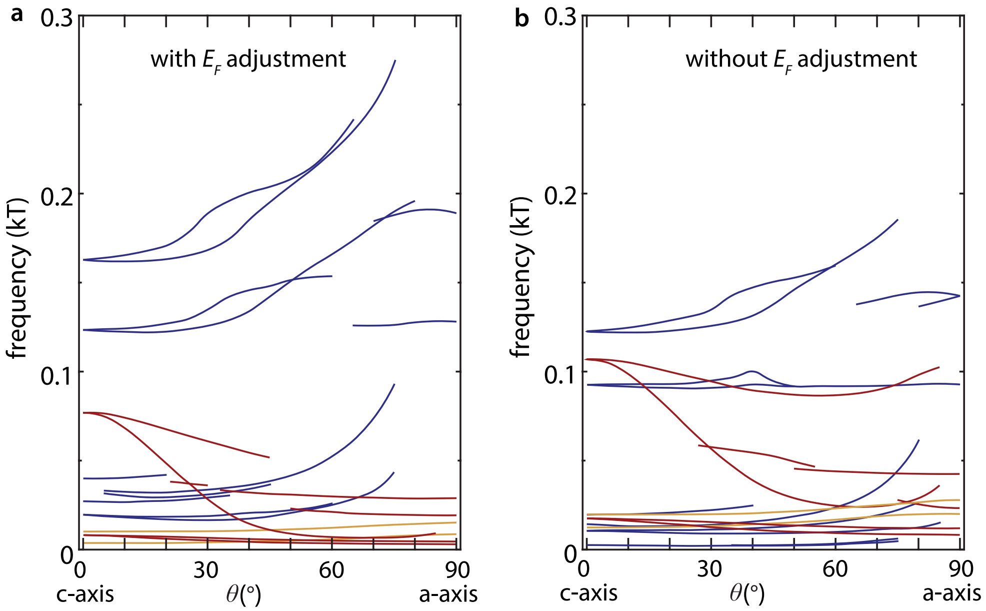

Supplementary Figure 8: **a,b,** Theoretically predicted angle dependent oscillation frequency of γ pockets, in the SP phase ($m \parallel c$) of $EuGa_4$, with and without E_F adjustment, respectively.

Since the γ pockets arise from the bands that form the red/blue Weyl NRs (see Fig. 1f,i in the main text), we conclude that the energy window of the red/blue NR states are $165 - 195$ meV, which is quite small considering it spans the whole $k_z = \pm 2\pi/c$ plane of the Brillouin zone.

In Supplementary Fig. 7d, we show all the possible extremal cross-sectional orbits associated with the γ pockets in the SP phase of $EuGa_4$ when the field is parallel to the c-axis, based on the DFT calculations. The blue ones are the hole pockets, while the red and oranges ones are the electron pockets. The size of the extremal orbits after E_F adjustment for the blue pockets are: 19.7 T, 27.2 T, 39.8 T, 123 T, and 163 T. Those for the red pockets are 8.1 T and 77 T. Those for the orange pockets are 3.6 T and 10.1 T. Their angle dependent QO frequencies are plotted in Supplementary Fig. 8a. For reference, we also show the angle dependent QO frequencies without any E_F adjustment in Supplementary Fig. 8b.

Supplementary Note 8. Cyclotron effective mass of the γ pockets

The measured temperature dependent QOs are presented in Supplementary Fig. 9. The L-K fit based on four frequency components are also presented on the top of experimental data. The temperature dependent amplitude of each oscillation component is presented in Fig. 3g in the main text.

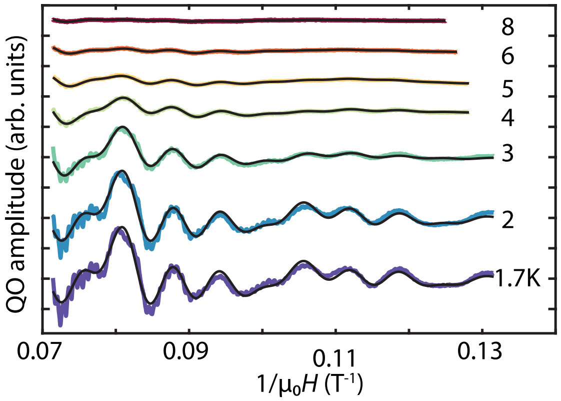

Supplementary Figure 9: Quantum oscillation measured at different temperatures from 1.7 K to 8 K when $H \parallel c$ ($\theta = 0°$). The curves are purposely vertically shifted for better visualization. Note that the solid black lines are L-K fits, based on the four frequency components of $\gamma_1 = 30$ T, $\gamma_2 = 77$ T, $\gamma_3 = 125$ T, and $\gamma_4 = 163$ T, as discussed in the main text.

Theoretically, the effective mass, m_{eff}, is expressed as the derivative of the cyclotron orbit area A with respect to the energy E [13]:

$$m_{\text{eff}} = \frac{\hbar^2}{2\pi}\frac{\partial A}{\partial E}$$

.

Based on the DFT band structures, $\frac{\partial A}{\partial E}$ can be readily calculated with a small variation of E_F, thus providing a way to evaluate the effective mass for each FS pocket in the single-particle frame without correlation effects. As discussed in the main text and Section 6, the QO frequency γ_4 is associated with the outer blue γ pocket (Supplementary Fig. 5c), while the nature of the measured lower frequencies is not clearly identified. For analysis, we have calculated the effective masses of all the possible extremal cyclotron orbits that were illustrated in Supplementary Fig. 7d. For the two types of the extremal orbits of the outer blue pocket, the effective masses are $0.14m_e$ and $0.075m_e$, where m_e is the mass of an electron. For the three types of the extremal orbits of the inner blue pocket, the effective masses are $0.11m_e$, $0.062m_e$, and $0.064m_e$. For the outer and inner red pockets, the effective masses are $0.18m_e$ and $0.05m_e$, respectively. For the outer and inner orange pockets, the effective masses are $0.03m_e$ and $0.02m_e$, respectively. Apparently, they are all smaller than those ($0.68 - 0.76m_e$) determined from experiments. We thus conclude that electronic correlation plays a role for the enhanced effective masses.

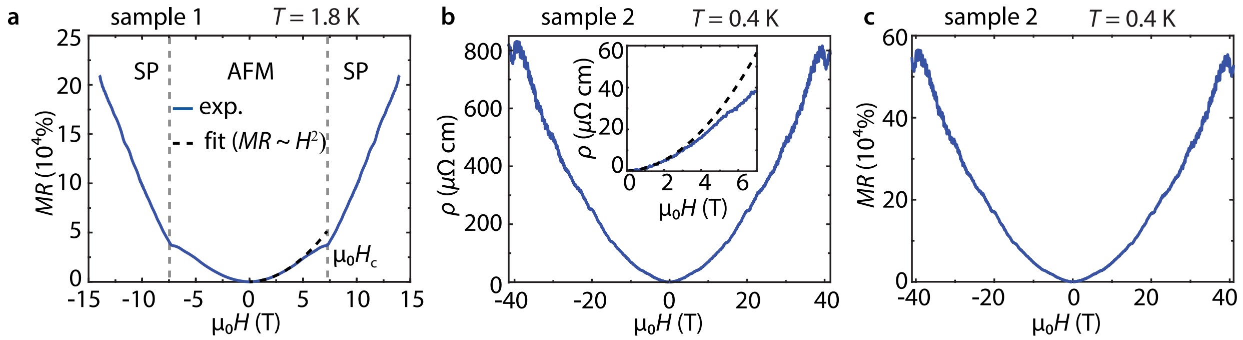

Supplementary Figure 10: MR behaviors of $EuGa_4$. **a**, MR curves measured in lab magnetometer up to 14 T on sample 1. H^2 fit is performed from 0 to 3.5 T. The AFM-SP phase transition is marked by $\mu_0 H_c$. **b**, Field dependent resistivity from -41.5 T to 41.5 T at 0.4 K measured on sample 2. Inset shows the H^2 fit to the low-field resistivity up to 2.5 T. **c**, The high-field MR curve converted from (b).

Supplementary Note 9. Large, non-saturating MR in $EuGa_4$

We performed field-dependent in-plane resistivity measurement on $EuGa_4$ both in our lab magnetometer (up to 14 T at 1.8 K) and using the high-field facility (up to 41.5 T at 0.4 K). Two samples are involved in the measurements. The measured resistivity in the positive- and negative-field sweeps is shown in Supplementary Fig. 10a,b. The MR curves on both samples show signatures of AFM-SP transition; the transition field is marked by arrows in Figs. 4b,c. To avoid possible sample heating issues from contacts at $T = 0.4$ K during the high-field measurement, we intentionally applied a small current $j = 3$ mA, which results in a low signal-noise-ratio at the low-field regime. In particular, the zero-field resistance reading has a large variation. Since the low-field MR behavior can be nicely described by an H^2 relation, an H^2 fit to the low-field region (inset, Supplementary Fig. 10b) is performed and the zero-field resistivity is obtained by the fit. Accordingly, we obtain the MR curve for the high-field measurement, as shown in Supplementary Fig. 10c. At 0.4 K and ~ 40 T, the MR exceeds 0.5×10^6%. Note that the resistivity dip at 40 T is from quantum oscillation. The MR measurements from our lab magnetometer and high-field facility show clear deviation from the H^2 relation below H_c. The field locations where the deviation occurs are at ~ 3.5 T and ~ 2 T for the former and latter measurements, respectively. The difference might be due to a slight difference in the sample quality.

Supplementary Note 10. MR value of $EuGa_4$ compared to that of other known topological semimetals

Supplementary Table 1 lists the non-magnetic and magnetic topological semimetals and their MR values that are included for comparison with $EuGa_4$. The plot is shown in Fig. 4e.

Supplementary Table 1: List of TSMs with their respective MR, temperature (T) and field ($\mu_0 H$) conditions.

Compound	MR (%)	T (K)	$\mu_0 H$ (T)	Reference
Na_3Bi	529	4.5	9	[14]
TaAs	70,000	1.8	9	[15]
Cd_3As_2	133,600	5	9	[16]
WTe_2	452,700	4.5	14.7	[17]
NbP	850,000	1.85	9	[18]
$Co_3Sn_2S_2$	53	2	14	[19]
Fe_3Sn_2	88.6	0.6	14	[20]
MnBi	250	2	9	[21]
PrAlSi	314	2	9	[22]
CeAlGe	6	2	7	[23]
NdPtBi	75	2	9	[24]
HoPtBi	122	2	14	[25]
GdPtBi	150	2	9	[26]
FeSn	224	0.4	14	[27]
$SrMnBi_2$	291	2	14	[5]
TbPtBi	392	2	14	[25]
NdAlSi	454	2	14	[28]
$YbMnBi_2$	573	2	9	[7]
$EuGa_4$	210,000	2	14	This work.

Supplementary Note 11. Carrier density of $EuGa_4$

The DFT predicted Fermi surface reasonably well describes the measured angle dependent quantum oscillation data, except slight overestimates of the outer cross-sections of the spin-split β-pockets (electron), and the high-angle ($\theta > 60°$) cross-sections of the α-pockets (hole) (Fig. 3c in the main text). Overall, we expect an overestimate of the carrier density by the DFT calculation. With the experimental quantum oscillation data, we are able to improve the accuracy. To this end, we

constructed the tight-binding model Hamiltonian of $EuGa_4$ in the SP phase according to the DFT calculation result. We then selectively adjust the energy of the bands gently to reproduce the experimentally measured quantum oscillation frequencies. Essentially, we projected the Bloch wavefuctions onto maximally localized Wannier functions (MLWFs) [29], and the model Hamiltonian was constructed from the MLWFs overlap matrix. In Supplementary Figs. **11**a–c, we show the FS plots of the α- and $\beta-$pockets after the band adjustment. By comparing these FS pockets with the ones (Supplementary Fig. **5**) without band adjustment, one can see that the FS maintains the same morphology except slight shrinking or distortion. We show the angle dependent quantum oscillation data from experiment and theory, before and after the band adjustment in Supplementary Figs. **11**d,e.

By comparing panel (d) and panel (e) in Supplementary Fig. **11**, one can observe that the band adjustment treatment has indeed quantitatively improved the accuracy of the theory calculated Fermi surface. We thus calculate the carrier density associated with each pocket and present the results in Supplementary Table **2**. The total electron and hole carrier density are determined to be 1.01×10^{21}cm^{-3} and 0.92×10^{21}cm^{-3}, respectively. The ratio is thus determined to be $n_e/n_h = 1.10$ after the band adjustment. For comparison, $n_e/n_h = 1.40$ before any band adjustment.

Supplementary Table 2: Carrier density of $EuGa_4$ in the SP phase.

Fermi surface	carrier type	carrier density before band adjustment ($\times 10^{20}$cm^{-3})	carrier density after band adjustment ($\times 10^{20}$cm^{-3})
α	hole	3.79<br>4.62	3.65<br>4.05
γ (blue)	hole	0.33<br>0.60	0.58<br>0.92
β	electron	5.87<br>7.22	4.43<br>5.58
γ (red)	electron	0.02<br>0.13	<0.01<br>0.07
γ (orange)	electron	<0.01<br>0.01	<0.01<br><0.01

Based on the angle dependent quantum oscillation data (Supplementary Fig. **11**e), we can evaluate the errors of n_e and n_h. For the $\alpha-$FS pockets, the spin splitting is clearly resolved by the measured two branches of quantum oscillation frequencies. In the meanwhile, the spin-splitting

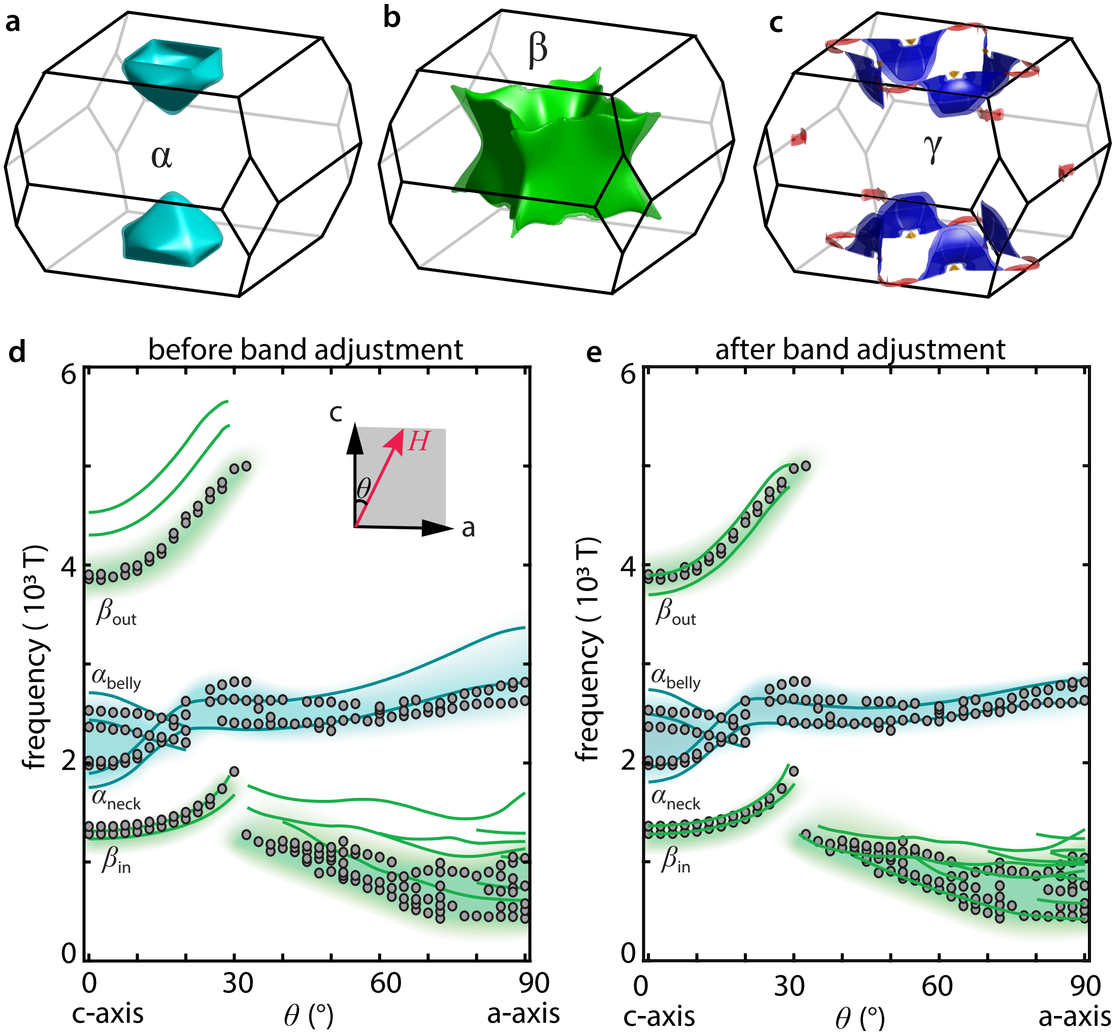

Supplementary Figure 11: **a–c** FS of $EuGa_4$ after band adjustment in the tight-binding model calculations. **d,e** The angle dependent quantum oscillation data with theoretical prediction before and after the band adjustment. Note that panel (d) is reproduced from Fig. 3c in the main text for the convenience of comparison.

also causes a difference in the carrier density associated with the two spin-split pockets. The difference is $0.4 \times 10^{20}\text{cm}^{-3}$ (see Supplementary Table 2). Consequently, the error of carrier density associated with the $\alpha-$pockets must be smaller than half of the difference, which gives $\Delta n_h\,(\alpha) < 0.4 \times 10^{20}\text{cm}^{-3}/2 = 0.2 \times 10^{20}\text{cm}^{-3}$. For the torus-shaped $\beta-$FS, the inner (β_{in}) and outer (β_{out}) extremal orbits appear below $\sim 30°$. For the β_{in} orbits, the experiment and theory match really well. For the β_{out} orbits, the lower- and higher-branches of the oscillation frequencies are slightly smaller and higher, respectively, than the experimental ones. On average, $n_h\,(\beta)$ estimated from theory should be close to the experimental one. If we take an extremely conservative estimate, the error can be calculated as half of the difference in the carrier density: $\Delta n_h\,(\beta) < 1.15 \times 10^{20}\text{cm}^{-3}/2 = 0.58 \times 10^{20}\text{cm}^{-3}$. Compared to the $\alpha-$ and $\beta-$pockets, the $\gamma-$pockets are much smaller in volume. Therefore, $\Delta n_h\,(\gamma)$ should be considerably smaller than $\Delta n_h\,(\alpha)$ and $\Delta n_h\,(\beta)$. Based on the analysis above, we conclude that the dominant source of error in determining n_e/n_h is on the β-pockets. The error bar is thus determined: $\Delta(n_e/n_h) < 6\%$.

Overall, the ratio of electron and hole carrier density in the SP phase of $EuGa_4$ is $n_e/n_h = 1.10 \pm 0.06$. We conclude that the close electron-hole carrier density may play a role for the large MR at low fields, but is not close enough to unity to achieve nonsaturating MR up to ~ 40 T. We note that the carrier density evaluated this way is more accurate than Hall measurements based on isotropic two-band model, because of the intrinsic FS anisotropy and multiband nature in $EuGa_4$.

Supplementary Note 12. Magnetotransport theory of Weyl nodal-ring semimetals

Consider the following model Hamiltonian for a nodal-ring semimetal:

$$\hat{h}(\mathbf{k}) = v_z k_z \hat{\sigma}_2 + \xi_{\mathbf{k}_\parallel} \hat{\sigma}_3 - \mu, \qquad \xi_{\mathbf{k}_\parallel} = \frac{k_\parallel^2}{2m} - E_M, \tag{1}$$

where $k_\parallel = \sqrt{k_x^2 + k_y^2}$, $\hat{\sigma}_i$ is Pauli matrix in the orbital space, E_M is an energy scale controlling the radius of the nodal ring, m is a parameter controlling the in-plane effective mass, and μ is a parameter controlling the chemical potential. The eigenenergies are given by

$$\pm\,\varepsilon_{\mathbf{k}} = \pm\sqrt{(v_z k_z)^2 + \xi_{\mathbf{k}_\parallel}^2} - \mu. \tag{2}$$

Evidently, when $k_x^2 + k_y^2 = 2mE_M$ and $k_z = 0$, the system is gapless, forming a nodal ring with a radius $k_0 = \sqrt{2mE_M}$. The corresponding band structure is shown in the inset of Supplementary Fig. 14a.

For this Weyl nodal ring model, the Berry curvature for the conduction band is known to be

$\mathbf{\Omega}_{+,\mathbf{k}} = \pi\,\delta(k_0 - k_\parallel)\,\delta(k_z)\,\hat{\phi}$, which is concentrated along the nodal ring [30]. The expression for the hole band is similar. While such non-trivial topology can give rise to an interesting anomalous transverse current [30], it is not our primary concern here. This is because the electric field $\mathbf{E} \parallel \hat{x}$ and $\mathbf{B} \parallel \hat{z}$ in the experiment and our main focus is on computing σ_{xx} and σ_{xy}.

A. Kinetic theory for magnetoconductivity

In the following calculation, we assume $\mu > 0$. In the presence of space-time uniform external electric and magnetic fields, the kinetic equation governing the distribution function of electrons in the linear response regime is given by [31, 32]

$$-\,eE\,v^x_{\mathbf{k}}\,\frac{\partial f_{e,0}}{\partial \varepsilon} - eB(v^y_{\mathbf{k}}\,\partial_{k_x} - v^x_{\mathbf{k}}\,\partial_{k_y})\,\delta f_e(\mathbf{k}) = I_{\mathsf{coll}}[f_e(\mathbf{k})], \tag{3}$$

where $\mathbf{v}_{\mathbf{k}} = \boldsymbol{\nabla}_{\mathbf{k}}\varepsilon_{\mathbf{k}}$ is the velocity of electrons, $f_{e,0}(\varepsilon) = 1/[1 + \exp(\varepsilon/T)]$ is the Fermi Dirac distribution function, T denotes temperature, and $\delta f_e(\mathbf{k})$ describes the deviation from equilibrium. Note that because of the orientation of the external fields ($\mathbf{B} \parallel \hat{z}$) in the experiment, the Berry curvature does not enter the kinetic equation [32]. For quenched onsite impurity potential disorder, the collision integral is given by [33]:

$$I_{\mathsf{coll}}[f_e] = 2\pi\lambda_{\mathsf{imp}} \int\limits_{\mathbf{q}} \frac{1 + \hat{d}_{\mathbf{k}} \cdot \hat{d}_{\mathbf{q}}}{2}\, 2\pi\delta(\varepsilon_{\mathbf{q}} - \varepsilon_{\mathbf{k}})\,[f_e(\mathbf{q}) - f_e(\mathbf{k})], \tag{4}$$

where $\int_{\mathbf{q}} = \int \frac{d^3q}{(2\pi)^3}$, $\mathbf{d}_{\mathbf{k}} = (0, v_z k_z, \xi_{\mathbf{k}_\parallel})$, λ_{imp} is a parameter controlling the disorder strength, and the Dirac delta function $\delta(\varepsilon_{\mathbf{q}} - \varepsilon_{\mathbf{p}})$ imposes energy conservation. The factor $(1 + \hat{d}_{\mathbf{p}} \cdot \hat{d}_{\mathbf{q}})/2$ arises due to the matrix structure of the Hamiltonian and accounts for the enhancement of forward scattering.

The kinetic equation can be solved using the *ansatz* $\delta f_e(\mathbf{k}) = eE(\partial_\varepsilon f_{e,0})\mathbf{v}^{\parallel}_{\mathbf{k}} \cdot \boldsymbol{\kappa}(k)$, where $\boldsymbol{\kappa} = (\kappa_x, \kappa_y)$ is an undetermined function depending only on the norm of $\mathbf{k}$, $\phi = \tan^{-1}(k_y/k_x)$, and $k_\parallel = \sqrt{k_x^2 + k_y^2}$. Performing the $\mathbf{q}$ integral in $I_{\mathsf{coll}}[f_e]$ and solving the kinetic equation, we find

$$\begin{bmatrix} \kappa_x(k) \\ \kappa_y(k) \end{bmatrix} = \frac{\tau_{\mathsf{tr}}(\mathbf{k})}{1 + [\omega_{c,\mathsf{eff}}(\mathbf{k})\tau_{\mathsf{tr}}(\mathbf{k})]^2} \begin{bmatrix} 1 \\ \omega_{c,\mathsf{eff}}(\mathbf{k})\tau_{\mathsf{tr}}(\mathbf{k}) \end{bmatrix}, \tag{5}$$

where $\omega_{c,\mathsf{eff}}(\mathbf{k}) = \omega_c\,\xi_{\mathbf{k}_\parallel}/\varepsilon_{\mathbf{k}}$ and $1/\tau_{\mathsf{tr}}(\mathbf{k}) = (m\pi\lambda_{\mathsf{imp}}/v_z)\varepsilon_{\mathbf{k}}$. Physically, $\omega_{c,\mathsf{eff}}$ represents the effective cyclotron frequency of electrons. Interestingly, $\omega_{c,\mathsf{eff}}$ flips sign across the nodal ring. Meanwhile, $1/[2\tau_{\mathsf{tr}}(\mathbf{k})]$ represents the impurity scattering rate for transport. For small T, $\varepsilon_{\mathbf{k}}$ is pinned at the chemical potential μ and thus the transport rate is approximately a constant.

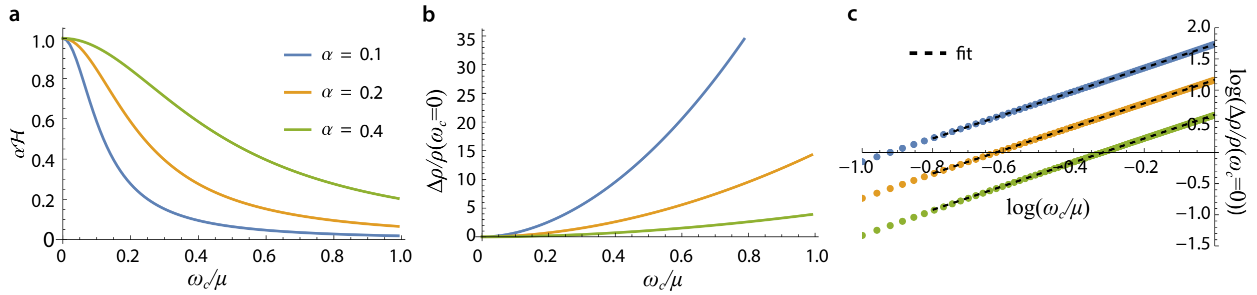

Supplementary Figure 12: **a**, $\mathcal{H}(\omega_c, \mu, \alpha)$ as a function of field at three different impurity strengths. Note that in the plot the field is in a normalized unit, ω_c/μ. Here α is a parameter controlling the impurity strength, ω_c is the cyclotron frequency and $\mu > 0$ is the chemical potential. **b**, $\Delta\rho_{xx}/\rho_{xx}$ as a function of field. **c**, Same as (b), but in $\log - \log$ scale. The linear fit (black dashed lines) reveals $\Delta\rho_{xx}/\rho_{xx} \sim \omega_c^\beta$, with $\beta \simeq (1.87, 1.88, 1.85)$ for $\alpha = (0.1, 0.2, 0.4)$ respectively. In all these panels, we have set $\mu = 1$.

For $\mu > 0$ and $T \to 0$, the current along $\mathbf{E} \parallel \hat{x}$ is solely contributed by electrons,

$$J_e^x = -e \int_{\mathbf{k}} v_{\mathbf{k}}^x \, \delta f_e(\mathbf{k}) = \sigma_{xx}(\omega_c) E, \tag{6}$$

where

$$\sigma_{xx}(\omega_c) = \sigma_0 \, \mathcal{H}(\omega_c, \mu, \alpha), , \qquad \mathcal{H}(\omega_c, \mu, \alpha) = 2\,\alpha \, \frac{\mu^2}{\omega_c^2} \left(1 - \frac{\alpha}{\sqrt{\alpha^2 + (\omega_c/\mu)^2}} \right). \tag{7}$$

Here $\alpha = m\pi\lambda_{\mathsf{imp}}/v_z$ is a dimensionless quantity characterizing the disorder strength and $\sigma_0 = \frac{e^2 E_M}{4\pi v_z}$ has the dimension of conductivity. Note that for $\omega_c \to 0$, $\mathcal{H}(\omega_c \to 0, \mu, \alpha) \to \alpha^{-1}$ and we recover the conductivity at $B = 0$: $\sigma_{xx}(\omega_c = 0) = \sigma_0/\alpha$. The ω_c dependence of the function $\mathcal{H}$ is shown in Supplementary Fig. 12a.

Meanwhile, the Hall current is written as:

$$J_e^y = -e \int_{\mathbf{k}} v_{\mathbf{k}}^y \, \delta f_e(\mathbf{k}) = e^2 E \int_{\mathbf{k}} \left(-\frac{\partial f_{e,0}}{\partial \varepsilon_{\mathbf{k}}} \right) \left(\frac{\xi_{\mathbf{k}_\parallel}}{\varepsilon_{\mathbf{k}}} \frac{k_\parallel}{m} \sin\phi \right)^2 \tau_{\mathsf{tr}}(\mathbf{k}) \frac{\omega_{c,\mathsf{eff}}(\mathbf{k})\tau_{\mathsf{tr}}(\mathbf{k})}{1 + [\omega_{c,\mathsf{eff}}(\mathbf{k})\tau_{\mathsf{tr}}(\mathbf{k})]^2}, \tag{8}$$

which is approximately 0 for large E_M, because $\omega_{c,\mathsf{eff}}(\mathbf{k})$ flips sign across the nodal ring as the velocity $\mathbf{v}_{\mathbf{k}}$ is reversed, implying that $\sigma_{xy} \simeq 0$. As we show below, the negligible Hall conductivity has important consequences for the nonsaturating MR observed in a Weyl nodal-ring system.

Recall that in a normal one-band metal, the conductivity tensor is given by [34]

$$\hat{\sigma}^{\mathsf{metal}} = \begin{bmatrix} \sigma_{xx}^{\mathsf{metal}} & \sigma_{xy}^{\mathsf{metal}} \\ -\sigma_{xy}^{\mathsf{metal}} & \sigma_{xx}^{\mathsf{metal}} \end{bmatrix}, \tag{9}$$

where

$$\sigma_{xx}^{\mathsf{metal}} = \frac{1}{1+(\omega_c\tau_{\mathsf{el}})^2}\sigma_{\mathsf{Drude}}, \qquad \sigma_{xy}^{\mathsf{metal}} = \frac{\omega_c\tau_{\mathsf{el}}}{1+(\omega_c\tau_{\mathsf{el}})^2}\sigma_{\mathsf{Drude}}, \tag{10}$$

In the equations, σ_{Drude} is the Drude conductivity and τ_{el} is the elastic scattering rate due to disorder. The resistivity tensor is obtained by matrix inverse operation: $\hat{\rho}^{\mathsf{metal}} = [\hat{\sigma}^{\mathsf{metal}}]^{-1}$. It leads to $\rho_{xx}^{\mathsf{metal}} = \sigma_{\mathsf{Drude}}^{-1}$, which has no B field dependence at all. However in Weyl nodal ring semimetal systems, the velocity sign flip leads to neglibily small Hall conductivity ($\sigma_{xy} \simeq 0$). Thus the transverse resistivity can be simply obtained by $\rho_{xx} \simeq 1/\sigma_{xx}$, implying a *nonsaturating* MR behavior which does not require perfect compensation of electrons and holes.

In Supplementary Fig. 12b, we plot $\Delta\rho_{xx}/\rho_{xx} = 1/(\alpha\mathcal{H}) - 1$ as a function of ω_c/μ for various impurity strength controlled by α. The nonsaturating behavior of ρ_{xx} is clear. The field dependence of $\Delta\rho_{xx}/\rho_{xx}$ can be fitted well with a power function, i.e. $\Delta\rho_{xx}/\rho_{xx} \sim \omega_c^{\beta}$, as shown in the log-log plot in Supplementary Fig. 12c. The linear fittings reveal that the exponent, β, is approximately in the range between 1.8 and 1.9.

Note that the non-saturating MR behavior illustrated above is solely due to the NR band structure and arises from the orbital degree of freedom. In the presence of spin degeneracy in nonmagnetic systems, the conductivity from both spin species simply adds up. For the case of a small Zeeman splitting, their contributions are roughly the same. Since the Hall conductivity of both spin channels remains low for a relatively large E_M, the non-saturating MR behavior is expected.

The discussion above considers a NR system without a gap. For a generic material system without magnetism or in the absence of symmetry protection, the NR can be gapped. Below we show that the non-saturating MR behavior holds even when a small gap is opened at the NR. We introduce a small gap term $\delta\hat{h} = \Delta\hat{\sigma}_1$ to the original model in Supplementary Eq. (1). The chemical potential is assumed to sit in the conduction band ($\mu > \Delta$). The eigenenergies are then expressed as:

$$\pm\,\varepsilon'_{\mathbf{k}} = \pm\sqrt{(v_z k_z)^2 + \xi_{\mathbf{k}_\parallel}^2 + \Delta^2} - \mu. \tag{11}$$

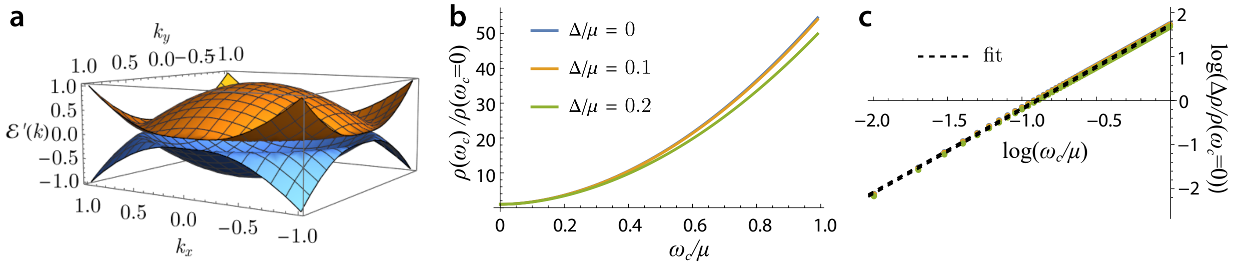

Supplementary Figure 13: **a**, Plot of the band structure described by $\varepsilon'_{\mathbf{k}}$ at $k_z = 0$ in the presence of a gap term $\delta\hat{h}$. **b**, Plot of the field dependence of the magnetoresistivity ρ_{xx} in the presence of the gap Δ. Here, $\alpha = 0.1$ and $\mu = 1$. **c**, Same as (b), but in log-log scale. The linear fit (black dashed line) reveals $\rho_{xx} \sim \omega_c^{\beta}$, with β slightly below 1.90 for three different gap parameters.

At $k_z = 0$, the original nodal ring located at $k_x^2 + k_y^2 = 2mE_M$ is now gapped out with a band gap of size 2Δ [see Supplementary Fig. 13(a)]. $\mathbf{d}_{\mathbf{k}}$ in Supplementary Eq. (4) is updated as $\mathbf{d}'_{\mathbf{k}} = (\Delta, v_z k_z, \xi_{\mathbf{k}_\parallel})$. The impurity collision integral can be evaluated in a similar fashion at small T. The gap term Δ only renormalizes the impurity strength λ_{imp} in the collision integral to $\lambda'_{\mathsf{imp}} = \lambda_{\mathsf{imp}}(1 + \Delta^2/\mu^2)$. Hence,

$$\begin{bmatrix} \kappa'_x(k) \\ \kappa'_y(k) \end{bmatrix} = \frac{\tau'_{\mathsf{tr}}(\mathbf{k})}{1 + [\omega_{c,\mathsf{eff}}(\mathbf{k})\tau'_{\mathsf{tr}}(\mathbf{k})]^2} \begin{bmatrix} 1 \\ \omega_{c,\mathsf{eff}}(\mathbf{k})\tau'_{\mathsf{tr}}(\mathbf{k}) \end{bmatrix}, \tag{12}$$

where $1/\tau'_{\mathsf{tr}}(\mathbf{k}) = (m\pi\lambda'_{\mathsf{imp}}/v_z)\varepsilon'_{\mathbf{k}}$.

Following Supplementary Eq. (6), the current along x is

$$J_e^x \simeq \frac{e^2 E}{(2\pi)^2} \int d\xi_{\mathbf{k}_\parallel} \int dk_z \left(-\frac{\partial f_{e,0}}{\partial \varepsilon'_{\mathbf{k}}}\right) \left(\frac{\xi_{\mathbf{k}_\parallel}}{\varepsilon'_{\mathbf{k}}}\right)^2 E_M \frac{\frac{m\pi\lambda'_{\mathsf{imp}}}{v_z}\varepsilon'_{\mathbf{k}}}{\left(\omega_c \frac{\xi_{\mathbf{k}_\parallel}}{\varepsilon'_{\mathbf{k}}}\right)^2 + \left(\frac{m\pi\lambda'_{\mathsf{imp}}}{v_z}\varepsilon'_{\mathbf{k}}\right)^2}. \tag{13}$$

Evaluating the integrals for small T, we have

$$J_e^x \simeq \sigma'_0\, \mathcal{H}'(\omega_c, \mu, \alpha', \Delta) E, \tag{14}$$

where

$$\mathcal{H}'(\omega_c, \mu, \alpha', \Delta) = 2\alpha'\,\gamma^{-1}\,\frac{\mu^2}{\omega_c^2}\left[1 - \frac{\alpha'}{\sqrt{\alpha'^2 + \gamma\left(\frac{\omega_c}{\mu}\right)^2}}\right], \qquad \gamma = \frac{\mu^2 - \Delta^2}{\mu^2}, \tag{15}$$

$\sigma'_0 = \gamma\sigma_0$ and $\alpha' = m\pi\lambda'_{\mathsf{imp}}/v_z$. As $\omega_c \to 0$, $\mathcal{H}'(\omega_c \to 0, \mu, \alpha', \Delta) \to 1/\alpha'$.

For large E_M, J_e^y is approximately 0, since $\omega_{c,\mathsf{eff}}(\mathbf{k})$ still flips the sign across the nodal ring, despite the presence of the gap term Δ. Thus, the transverse resistivity is still given by $\rho_{xx} \simeq 1/\sigma_{xx}$. As shown in Supplementary Fig. **13**(b), the gap Δ only causes mild changes in the field dependence of ρ_{xx}. Overall, ρ_{xx} still exhibits a power-law dependence in B with the exponent slightly smaller than the one without the gap, as illustrated in Supplementary Fig.**13**(c).

B. Quantum theory for magnetoconductivity

While the semiclassical theory can well describe the low-field MR behavior, its accuracy is undermined when the system enters the Landau quantized regime, especially when a large enough field is applied so that the system approaches the quantum limit. We now turn into a fully quantum mechanical description of the MR using the Kubo formula.

As a first step, we calculate the Landau level spectrum. With the magnetic field $\mathbf{B} = B\hat{z}$, we employ the gauge $A_y = Bx,\ A_x = A_z = 0$, where $\mathbf{A}$ is the vector potential, and send $k_y \to k_y + eA_y$ in the Hamiltonian in Supplementary Eq. (1). We also account for the intrinsic non-flatness (energy variation) of the nodal ring by introducing an extra term $\delta\hat{h}(\mathbf{k}) = \lambda k_x^2/2m$ into the original model Hamiltonian in Supplementary Eq. (1). Here, $0 < \lambda < 1$ is a small parameter controlling the level of energy variation of the nodal ring. We treat such variation perturbatively for small λ. The eigenenergies at Landau level n is given by

$$\pm E_{n,k_z} = \pm\sqrt{(\varepsilon_n - E_M)^2 + (v_z k_z)^2} + \delta E_n, \qquad \varepsilon_n = \left(n + \frac{1}{2}\right)\omega_c, \qquad \delta E_n \simeq \lambda\omega_c \frac{2n+1}{4}, \tag{16}$$

where δE_n is the energy shift due to $\delta\hat{h}$ at order $\mathcal{O}(\lambda)$. The corresponding eigenstates can be expressed in terms of the Hermite polynomials.

We compute the xx and xy components of the current-current correlation function following the standard procedures outlined in Refs. [35, 36]. In the presence of disorder, we assume that the eigenstates are approximately unchanged and introduce a constant self-energy Γ_B, describing phenomenologically the impurity scattering rate as inspired by the kinetic theory results. We confirm that our expressions reduce to the ones based on the kinetic theory in the semiclassical limit for small field B and weak disorder. Physically, the Kubo calculation accounts for the discreteness of the Landau levels and the smearing of the spectral function, in addition to the semiclassical motion of electrons.

Below we present the field dependence of the MR by numerically evaluating the Kubo expression. We now consider a practical E_M value with the energy scale comparable to that of the Weyl nodal rings that lead to the formation of the β pockets in $EuGa_4$ (Supplementary Fig. 5b), and a small λ so that the Fermi surface in this model forms a torus geometry. Under this condition, we have $\sigma_{xy} \ll \sigma_{xx}$ and $\rho_{xx} \simeq 1/\sigma_{xx}$. In the numerical calculation, we summed over $n_{\mathsf{max}} = 50000$ Landau levels to ensure convergence. The results for a representative set of parameters are shown in Supplementary Fig. 14. Note that the x-axis in the figures is represented by a normalized field parameter, ω_c/E_M.

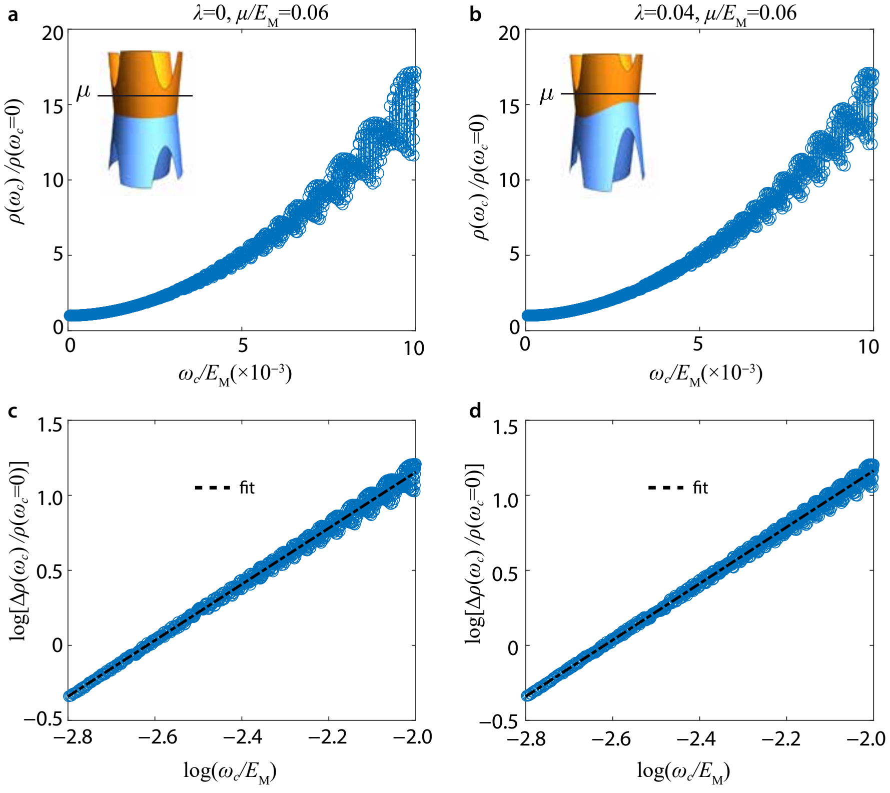

Supplementary Figure 14: Field dependent resistivity normalized by its zero-field value for (**a**) $\lambda = 0$ (flat nodal ring), (**b**) $\lambda = 0.04$ (non-flat nodal ring). In both cases, we set the chemical potential $\mu = 0.06E_M$ and impurity scattering rate $\Gamma_B = 10^{-3}E_M$. The insets show the corresponding band structure and chemical potential at $\omega_c = 0$ and $k_z = 0$. Note that for $\lambda > 0$, the energy of the nodal ring is shifted upward by an amount $\delta E/E_M \simeq \lambda/2$. **c,d** The corresponding log-log plots. The linear fit (black dashed lines) for the $\omega_c/E_M > 1.5 \times 10^{-3}$ data reveals $\Delta\rho_{xx}/\rho_{xx} \sim \omega_c^{\beta}$, with $\beta \simeq 1.90$ for both $\lambda = 0$ and 0.04).

We fix the chemical potential to be a positive value of $\mu = 0.06E_M$ and the impurity scattering

rate to be $\Gamma_B = 10^{-3}E_M$. The small scattering rate is justified given the high carrier mobility of the sample. We assume the field dependence of μ and Γ_B is weak and thus negligible. This is approximately valid at a finite $\mu > \omega_c$. The $\mu \to 0$ scenario is interesting but requires special attention [35, 37, 38]. We will leave it for future studies.

For the case of flat Weyl nodal ring ($\lambda = 0$) (Supplementary Fig. **14**a), ρ_{xx} shows an approximately quadratic field dependence at low fields ($\omega_c/E_M <\sim 1 \times 10^{-3}$) and does not exhibit obvious quantum oscillations, in agreement with the results from the kinetic theory. As the field further increases, the system enters the Landau quantized regime, and the resistivity demonstrates gradually enhanced oscillations due to the discreteness of the Landau level energy spectrum. With the existence of the inner and outer extremal cyclotron orbits of the nodal ring, the oscillations also show a beating pattern.

For the case of non-flat Weyl nodal ring ($\lambda > 0$), the results are shown in Supplementary Fig. **14**b. Here we assumed a relatively small energy variation, $\lambda = 0.04$. In this case, μ does not cross the nodal ring at $k_z = 0$ in the zero field limit (see the illustration in the inset). This scenario bears resemblance to the nodal rings that lead to the formation of β-pockets in $EuGa_4$. The field dependence is qualitatively the same as the $\lambda = 0$ case (Supplementary Fig. **14**a) except the change in the oscillation frequencies.

In both cases, we show the numerical calculations up to the field, $\omega_c/E_M = 0.01$, which is about $1/100$ of the field strength that is required for the system to reach quantum limit. This is comparable to the applied field strength in our measurements: the maximal measured field of ~ 40 T is about $1/100$ of the field strength that is required for the electrons with the β-pockets to reach quantum limit.

In Supplementary Figs. **14**c,d, we show the log-log plots of the MR curves. The linear fits of the high-field data (black dashed lines) indicate that the MR follows the power function $\Delta\rho_{xx}/\rho_{xx} \sim \omega_c^{\beta}$, with the exponent $\beta \simeq 1.90$ for both flat and nonflat Weyl nodal rings.

C. Discussion

We showed above the magnetotransport results based on semiclassical and quantum theory for a Weyl nodal ring system. we find that the nonsaturating MR naturally arises in a Weyl NR system, without the stringent requirement of perfect electron-hole carrier compensation [17, 39]. In fact, we only assumed one type of conducting carriers in our theoretical model. This unusual behavior

benefits from the negligibly small Hall conductivity, which derives from the sign reversal of the Fermi velocity across the nodal ring. We performed the Hall measurements on the high-quality $EuGa_4$ single crystal, and show the data in Supplementary Fig. **15**. The Hall resistivity, ρ_{yx}, is indeed significantly smaller than the transverse resistivity, ρ_{xx} (see Supplementary Fig. **10**). At 2 K and 14 T, ρ_{yx}/ρ_{xx} is only about 2%, which supports the treatment of $\rho_{xx} \simeq 1/\sigma_{xx}$ in our magnetotransport model.

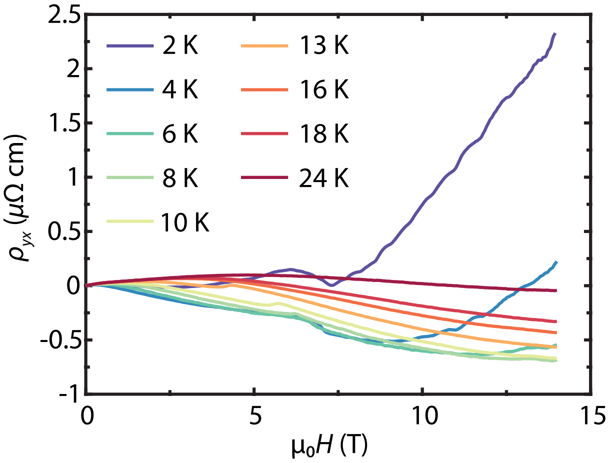

Supplementary Figure 15: Hall data measured at a series of temperatures from 2 K to 24 K.

We note that in our model, we did not consider the contribution of the small $\gamma-$pockets. Apparently, as $\mu \to 0$, electrons of the $\gamma-$pockets can be forced to occupy a few discrete Landau levels at much lower field than that of the bigger pockets. The magnetotransport properties in this scenario are interesting, but require special treatment [35, 37, 38], as we mentioned above. Nevertheless, it is safe to conclude that the contribution of the $\gamma-$pockets to the nonsaturating MR behavior is small, given the small fraction of the carriers from these pockets. This is in sharp contrast to the quantum magnetoresistance mechanism proposed by Abrikosov [35], where nonsaturating MR appears only when electrons occupy the lowest Landau level. Therefore, we establish Weyl nodal ring semimetals as a novel platform to host the nonsaturating MR. For nonmagnetic NR systems, SOC can induce a gap for the NR states. Our extended model suggests that, as long as the energy of the SOC-induced gap is much smaller than the binding energy of the nodal ring states, the nonsaturating MR behavior is preserved. Experimentally, a few non-magnetic topological nodal line semimetals, such as ZrSiS [40], ZrSiSe [41], and $BaAl_4$ [42], have been reported to show nonsaturating MR for fields up to 53 T, 62 T, and 35 T, respectively, although the mechanism responsible for the nonsaturating MR was not understood. Our work thus provides a unified explanation for the nonsaturating MR behavior for these systems.

Supplementary Note 13. Structural refinement from powder X-ray diffraction

Supplementary Table 3 provides the atomic positions for the structure of $EuGa_4$ from Rietveld refinement along with the corresponding lattice parameters. We carried out powder x-ray diffraction measurements and the corresponding diffraction peaks are shown in Supplementary Fig. 16.

Supplementary Table 3: Structural parameters for $EuGa_4$ at 300K. Space group I4/mmm (No. 139).

Atom	Wyckoff	Occupancy	x	y	z
Eu	2a	1	0	0	0
Ga1	4e	1	0	0	0.38388(17)
Ga2	4d	1	0	0.5	0.25
a = 4.39564(7) Å, c = 10.66121(19) Å					
R_{wp}=11.43%, R_{exp} = 7.04%					

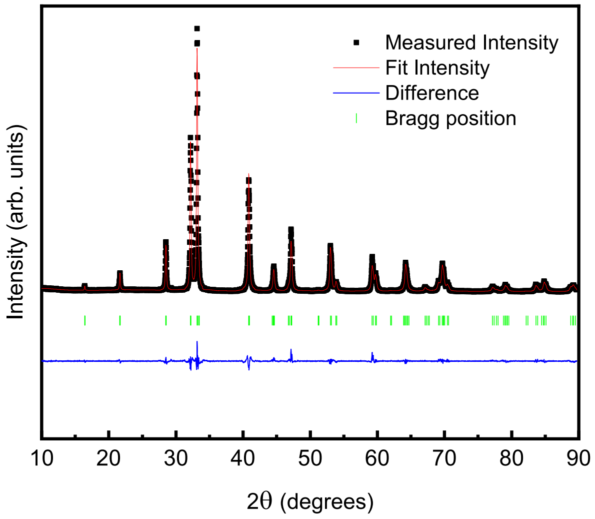

Supplementary Figure 16: Powder x-ray diffraction pattern of $EuGa_4$ taken at T = 300 K along with the Rietveld refinement (red line), the blue line is the difference between the measured and the fitted intensity while the green ticks correspond to the Bragg peak positions.

Supplementary References

1. Bzdušek, T., Wu, Q., Rüegg, A., Sigrist, M. & Soluyanov, A.A. Nodal-chain metals. *Nature* **538**, 75-78 (2016).

2. Chang, G. et al. Topological Hopf and chain link semimetal states and their application to Co_2MnGa. *Phys. Rev. Lett.* **119**, 156401 (2017).

3. Lee, G., Farhan, M.A., Kim, J.S. & Shim, J.H. Anisotropic Dirac electronic structures of $AMnBi_2$ (A= Sr, Ca). *Physical Review B* **87**, 245104 (2013).

4. Klemenz, S., Lei, S. & Schoop, L.M. Topological semimetals in square-net materials. *Annual Rev. of Mat. Res.* **49**, 185-206 (2019).

5. Park, J., et al. Anisotropic Dirac fermions in a Bi square net of $SrMnBi_2$. *Phys. Rev. Lett.* **107**, 126402 (2011).

6. Wang, J.K. et al. Layered transition-metal pnictide $SrMnBi_2$ with metallic blocking layer. *Phys. Rev. B* **84**, 064428 (2011).

7. Borisenko, S. et al. Time-reversal symmetry breaking type-II Weyl state in $YbMnBi_2$. *Nature* **10**, 3424 (2019).

8. Guo, Y.F. et al. Coupling of magnetic order to planar Bi electrons in the anisotropic Dirac metals $AMnBi_2$ (A= Sr, Ca). *Phys. Rev. B* **90**, 075120 (2014).

9. Hasan, M.Z. et al. Weyl, Dirac and high-fold chiral fermions in topological quantum matter. *Nat. Rev. Mat.* **6**, 784-803 (2021).

10. Burkov, A.A., Hook, M.D. and Balents, L. Topological nodal semimetals. *Phys. Rev. B* **84**, 235126 (2011).

11. Nakamura, A. et al. Magnetic and Fermi surface properties of $EuGa_4$. *J. of the Phys. Soc. of Japan* **82**, 104703 (2013).

12. Teicher, S.M. et al. 3D Analogs of Square-Net Nodal Line Semimetals: Band Topology of Cubic $LaIn_3$. *Chem. of Mat.* **34**, 4446-4455 (2022).

13. Shoenberg, D. *Magnetic oscillations in metals* (Cambridge university press, 2009).

14. Xiong, J. et al. Evidence for the chiral anomaly in the Dirac semimetal Na_3Bi. *Science* **350**, 413-416 (2015).

15. Huang, X. et al. Observation of the chiral-anomaly-induced negative magnetoresistance in 3D Weyl semimetal TaAs. *Phys. Rev. X* **5**, 031023 (2015).

16. Liang, T., Gibson, Q., Ali, M.N., Liu, M., Cava, R.J. and Ong, N.P., 2015. Ultrahigh mobility and giant magnetoresistance in the Dirac semimetal Cd_3As_2. *Nat. Mat.* **14**, 280-284 (2015).

17. Ali, M.N. et al. Large, non-saturating magnetoresistance in WTe_2. *Nature* **514**, 205-208 (2014).

18. Shekhar, C. et al. Extremely large magnetoresistance and ultrahigh mobility in the topological Weyl semimetal candidate NbP. *Nat. Phys.* **11**, 645-649 (2015).

19. Liu, E. et al. Giant anomalous Hall effect in a ferromagnetic kagome-lattice semimetal. *Nat. Phys.* **14**, 1125-1131 (2018).

20. Ye, L. et al. Massive Dirac fermions in a ferromagnetic kagome metal. *Nature* **555**, 638-642 (2018).

21. He, Y. et al. Large linear non-saturating magnetoresistance and high mobility in ferromagnetic MnBi. *Nat. Comm.* **12**, 4576 (2021).

22. Lyu, M. et al. Nonsaturating magnetoresistance, anomalous Hall effect, and magnetic quantum oscillations in the ferromagnetic semimetal PrAlSi. *Phys Rev. B* **102**, 085143 (2020).

23. Suzuki, T. et al. Singular angular magnetoresistance in a magnetic nodal semimetal. *Science* **365**, 377-381 (2019).

24. Shekhar, C. et al. Anomalous hall effect in weyl semimetal half-heusler compounds RPtBi (R= Gd and Nd). *Proc. of the Nat. Aca. of Sci.* **115**, 9140 (2018).

25. Pavlosiuk, O. Fałat, P., Kaczorowski, D. & Wiśniewski, P. Anomalous Hall effect and negative longitudinal magnetoresistance in half-Heusler topological semimetal candidates TbPtBi and HoPtBi. *APL Mat.* **8**, 111107 (2020).

26. Hirschberger, M. et al. The chiral anomaly and thermopower of Weyl fermions in the half-Heusler GdPtBi. *Nat. Mat.* **15**, 1161-1165 (2016).

27. Kang, M. et al. Dirac fermions and flat bands in the ideal kagome metal FeSn. *Nat. Mat.* **19**, 163-169 (2020).

28. Gaudet, J. et al. Weyl-mediated helical magnetism in NdAlSi. *Nat. Mat.* **20**, 1650-1656 (2021).

29. Mostofi, A.A. et al. A tool for obtaining maximally-localised Wannier functions. *Com. Phys. Comm.* **178**, 685-699 (2008).

30. Rui, W.B., Zhao, Y.X. & Schnyder, A.P. Topological transport in Dirac nodal-line semimetals. *Phys. Rev. B* **97**, 161113 (2018).

31. Kamenev, A. Field Theory of Non-Equilibrium Systems. (Cambridge University Press, 2011).

32. Xiao, D., Chang, M.C. & Niu, Q. Berry phase effects on electronic properties. *Rev. Mod. Phys.* **82**, 1959 (2010).

33. Xie, H.Y. & Foster, M.S. Transport coefficients of graphene: Interplay of impurity scattering, Coulomb interaction, and optical phonons. *Phys. Rev. B* **93**, 195103 (2016).

34. Ashcroft, N.W. and Mermin, N.D. Solid State Physics. (Cengage Learning, 1976)

35. Abrikosov, A.A. Quantum magnetoresistance. *Phys. Rev. B* **58**, 5 (1998).

36. Abrikosov, A.A. Galvanomagnetic phenomena in metals in the quantum limit. *Sov. Phys. JETP* **29**, 746 (1969).

37. Klier, J., Gornyi, I.V. & Mirlin, A.D. Transversal magnetoresistance in Weyl semimetals. *Phys. Rev. B* **92**, 205113 (2015).

38. Xiao, X., Law, K.T. & Lee, P.A. Magnetoconductivity in Weyl semimetals: Effect of chemical potential and temperature. *Phys. Rev. B* **96**, 165101 (2017).

39. Mondal, R. et al. Extremely large magnetoresistance, anisotropic Hall effect, and Fermi surface topology in single-crystalline WSi_2. *Phys. Rev. B* **102**, 115158 (2020).

40. Wang, X. et al. Evidence of both surface and bulk Dirac bands and anisotropic nonsaturating magnetoresistance in ZrSiS. *Adv. Elec. Mat.* **2**, 1600228 (2016).

41. Chiu, Y.C. et al. Origin of the butterfly magnetoresistance in a Dirac nodal-line system. *Phys. Rev. B* **100**, 125112 (2019).

42. Wang, K. et al. Crystalline symmetry-protected non-trivial topology in prototype compound $BaAl_4$. *npj Quan. Mat.* **6**, 28 (2021).
